# Integrated Multiomics Reveals Silencing of has_circ_0006646 Promotes TRIM21‐Mediated NCL Ubiquitination to Inhibit Hepatocellular Carcinoma Metastasis

**DOI:** 10.1002/advs.202306915

**Published:** 2024-02-15

**Authors:** Xin Hu, Guanrong Chen, Yingchen Huang, Qiyang Cheng, Jianyong Zhuo, Renyi Su, Chiyu He, Yichao Wu, Zhikun Liu, Beng Yang, Shuai Wang, Lijun Meng, Shusen Zheng, Di Lu, Qiang Wei, Jiayin Yang, Xuyong Wei, Ronggao Chen, Xiao Xu

**Affiliations:** ^1^ Zhejiang University School of Medicine Hangzhou 310058 China; ^2^ Key Laboratory of Integrated Oncology and Intelligent Medicine of Zhejiang Province Hangzhou 310006 China; ^3^ NHC Key Laboratory of Combined Multi‐organ Transplantation Hangzhou 310003 China; ^4^ The Fourth School of Clinical Medicine Zhejiang Chinese Medical University Hangzhou 310053 China; ^5^ Department of Hepatobiliary Surgery Beijing Chaoyang Hospital affiliated to Capital Medical University Beijing 100020 China; ^6^ Department of Hepatobiliary and Pancreatic Surgery Affiliated Hangzhou First People's Hospital Zhejiang University School of Medicine Hangzhou 310006 China; ^7^ Department of Hepatobiliary and Pancreatic Surgery Shulan (Hangzhou) Hospital Hangzhou 310022 China; ^8^ Department of Hepatobiliary and Pancreatic Surgery The First Affiliated Hospital Zhejiang University School of Medicine Hangzhou 310006 China; ^9^ Department of Liver Surgery Liver Transplantation Center West China Hospital of Sichuan University Chengdu 332001 China

**Keywords:** Circular RNA, Hepatocellular carcinoma, multi‐omics, Nucleolin, ubiquitination

## Abstract

Recent studies suggest that circular RNA (circRNA)‐mediated post‐translational modification of RNA‐binding proteins (RBP) plays a pivotal role in metastasis of hepatocellular carcinoma (HCC). However, the specific mechanism and potential clinical therapeutic significance remain vague. This study attempts to profile the regulatory networks of circRNA and RBP using a multi‐omics approach. Has_circ_0006646 (circ0006646) is an unreported circRNA in HCC and is associated with a poor prognosis. Silencing of circ0006646 significantly hinders metastasis in vivo. Mechanistically, circ0006646 prevents the interaction between nucleolin (NCL) and the E3 ligase tripartite motif‐containing 21 to reduce the proteasome‐mediated degradation of NCL via K48‐linked polyubiquitylation. Furthermore, the change of NCL expression is proven to affect the phosphorylation levels of multiple proteins and inhibit p53 translation. Moreover, patient‐derived tumor xenograft and lentivirus injection, which is conducted to simulate clinical treatment confirmed the potential therapeutic value. Overall, this study describes the integrated multi‐omics landscape of circRNA‐mediated NCL ubiquitination degradation in HCC metastasis and provides a novel therapeutic target.

## Graphical abstract

Has_circ_0006646, a metastasis‐associated circRNA that is upregulated in HCC cells and tissues, can prevent interaction between NCL and the E3 ligase TRIM21 to reduce the ubiquitination degradation of NCL. This study expands the understanding of the epigenetic regulatory background of HCC metastasis and provides an exploitable therapeutic strategy for clinical treatment.

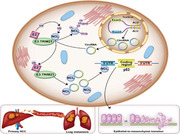

## 1 Introduction

Hepatocellular carcinoma (HCC), which accounts for most primary liver cancers (75−85%), is the sixth most commonly diagnosed cancer and has been acknowledged as the third leading cause of cancer‐related death worldwide.^[^ ^1^ ^]^ Despite the rapid development of systemic therapies such as chemotherapy, targeted drugs, and immunotherapy as well as surgical resection and liver transplantation (LT) over the past 70 years, the 5‐year survival rate of HCC is still not promising.^[^ ^2^, ^3^ ^]^

Metastasis and recurrence, or the consequences of resistance to therapy, jointly determine a fatal HCC result. Regarded as the systemic spread and proliferation of tumor cells throughout the body, metastasis was confirmed to largely contribute directly to the 5‐year survival rates of patients with solid tumors diagnosed with metastasis, which range from 5–30%.^[^ ^4^, ^5^ ^]^ Microvascular invasion (MVI), the presence of which impairs the effectiveness of surgery, is considered the inception phase of metastasis.^[^ ^6^ ^]^ As an initial step in metastasis, it is therefore particularly critical to decipher the molecular mechanism that actuate the occurrence of MVI.

With further research, gene mutations alone have been found to insufficiently account for tumorigenesis. The expanded understanding of epigenetic factors, such as noncoding RNA (ncRNA), has revealed another important role of tumorigenic processes.^[^ ^7^, ^8^, ^9^ ^]^ Formed by cis back‐splicing and circularization of introns, exons, and other transcripts, circular RNAs (circRNAs) constitute covalently closed single‐stranded loops and are considered as the emerging ncRNAs.^[^ ^10^, ^11^, ^12^, ^13^ ^]^ Accumulating evidence indicates that they are double‐edged swords, similar to Pandora's Box, in the process of HCC metastasis. For example, circRPN2 inhibits HCC metastasis through the miR‐183–5p/FOXO1 axis, while circASAP1 mediates tumor‐associated macrophage infiltration to promote pulmonary metastasis.^[^ ^14^, ^15^ ^]^ In addition, current studies of the roles of circRNAs in metastasis have concentrated more on the late stage of metastasis, such as lung metastasis, but neglected the early stage.^[^ ^15^ ^]^ Thus, the biological functions of circRNAs in MVI remain largely unknown, warranting further investigation.

Nucleolin (NCL), one of the most abundant proteins in the nucleolus, is associated with ribosomal RNA (rRNA) transcription and ribosome assembly.^[^ ^16^, ^17^ ^]^ Since NCL is a natural nucleic acid‐binding target, numerous studies have confirmed that NCL is highly relevant to tumor occurrence and development.^[^ ^18^, ^19^ ^]^ Not only protein levels but also various events closely related to NCL, such as posttranslational modification (PTM), changes in subcellular localization (especially in the cytoplasm and cell membrane), and the regulation of DNA damage, might influence the progression of tumors.^[^ ^18^, ^20^, ^21^, ^22^ ^]^ Although a few articles have reported the regulatory networks between ncRNAs and NCL in other diseases, the mechanism underlying a circRNA‐mediated change of NCL to modulate tumorigenesis (particularly in HCC) is still unclear and requires further exploration.^[^ ^23^, ^24^ ^]^

In this study, we demonstrated that has_circ_0006646 (circ0006646), an unreported and metastasis‐associated circRNA that was upregulated in HCC cells and tissues, was an independent risk factor for long‐term survival. Its presence could promote HCC invasion and migration in vivo and in vitro. Mechanistically, circ0006646 could prevent interaction between NCL and the E3 ligase tripartite motif‐containing 21 (TRIM21) to reduce the ubiquitination degradation of NCL. Finally, we further validated the therapeutic value of circ0006646 using patient‐derived tumor xenograft (PDX) models. In summary, our study expanded the understanding of the epigenetic regulatory background of HCC metastasis and provided an exploitable therapeutic strategy for clinical treatment.

## 2 Results

### 2.1 The Characteristics of circ0006646 in HCC

**Figure 1:**
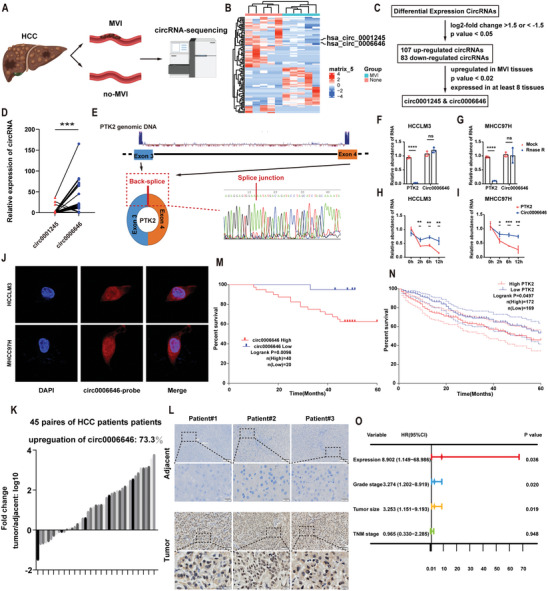
Circ0006646 was an up‐regulated circular RNA in HCC, indicating the worse prognosis. A) Sequencing process of CircRNAs. B) Heatmap showed the up‐regulated and down‐regulated circRNAs. C) Screening diagram of circRNAs. D) The expression level of circ00001245 and circ0006646 were detected by qRT‐PCR. E) The formation process of circ0006646 was shown. The structure was formed at the exon 3–4 by back splicing, which was confirmed by sanger sequencing. F,G) Total RNA was treated with RNase R, after which the expression level of circ0006646 and PTK2 in HCCLM3 (F) and MHCC97H (G) were assessed. H,I) Cells were treated with Actinomycin D. The expression level of circ0006646 and PTK2 in HCCLM3 (H) and MHCC97H (I) were measured at 0, 2, 6, and 12 h. J) Fluorescence in situ hybridization assay showed the subcellular localization of circ0006646. DAPI staining represented the location of the nucleus and the circ0006646 probe was labeled with cy3 (red). K) Circ0006646 expression ratios of “tumor tissues/adjacent tissues” were calculated. L) Representative ISH staining of paired tumor and adjacent tissues were shown. Scale bars, 100 and 20 µm, respectively. M) Kaplan–Meier analysis was conducted to assess overall survival rate in HCC patients according to the expression of circ0006646. N) The association between PTK2 expression level and prognosis of HCC patients was evaluated using TCGA database. O) COX regression model was used to define the independent prognostic factors of HCC. The hazard rate (HR) and 95% confidence intervals (CI) were shown in forest map. All experiments were performed in at least triplicate samples. Data were presented as the means ± SD. ^*^ *p* < 0.05, ^**^ *p* < 0.01, ^***^ *p* < 0.001, ^****^ *p* < 0.0001. Student's *t*‐test was used for normally distributed data and Mann–Whitney U test was used for skewed distribution data.

To identify potential circRNAs that promote HCC metastasis, we profiled global circRNA transcripts from 5 tissue pairs of HCC patients with and without MVI using RNA sequencing (**Figure** **1**A). Above all, 107 circRNAs were up‐regulated and 83 circRNAs were down‐regulated (selection criteria: log2‐fold change >1.5 or < −1.5, p < 0.05). Among them, circ0001245 and circ0006646 were chosen based on the criteria 1) upregulated in MVI‐present tissues, 2) p value < 0.02, and 3) expressed in at least 8 tissues (Figure 1B,C; Figure S1A and Table S4, Supporting Information). Subsequently, results of qRT‒PCR assays of HCC tissues showed that the expression level of circ0006646 was significantly higher (Figure 1D). Circ0006646 arises from the parent gene PTK2 and displays head‐to‐tail splicing of exons 3–4. A divergent primer was designed to amplify the back‐spliced site of PTK2 and Sanger sequencing was employed to validate the presence of spliced junctions within PTK2 (Figure 1E; Figure S1B, Supporting Information).

To demonstrate the stability of circ0006646, ribonuclease R (RNase R), an enzyme that digests linear transcript RNA, was used to treat the total RNA extracted from HCC cell line cells. Circ0006646 was more tolerant to RNase R than PTK2, suggesting that it had a special circular structure (Figure 1F,G; Figure S1C, Supporting Information). In addition, when RNA synthesis was inhibited by actinomycin D, the attenuation of PTK2 was significantly faster than that of circ0006646 (Figure 1H,I). RNA FISH and RNA fractionation (nuclear/cytosolic) assay demonstrated the higher abundance of circ0006646 in neclus (Figure 1J; Figure S1D, Supporting Information). In addition, the secondary structure of circ0006646 with the minimum free energy was predicted by RNAfold (http://rna.tbi.univie.ac.at//cgi‐bin/RNAWebSuite/RNAfold.cgi) (Figure S1E, Supporting Information). These results indicated that circ0006646 is a stable circular RNA in HCC.

### 2.2 Upregulation of circ0006646 is Associated with a Poor Prognosis of HCC

45 HCC and matched adjacent tissues from HCC cohort 1 were used to identify the expression of Circ0006646. It was found to be upregulated in most HCC tissues (73%) compared with para‐tumor tissues (Figure 1K; Figure S2A, Supporting Information). Similarly, analysis using the Gene Expression Profiling Interactive Analysis (GEPIA) database showed that the expression level of the parental gene PTK2 in HCC tissues was also higher than that in normal tissues. (Figure S2B–D, Supporting Information). This result was also verified by ISH assay (Figure 1L; Figure S2E, Supporting Information).

In addition, we evaluated the ability of circ0006646 to be a tumor marker. It showed that higher expression of circ0006646 was associated with higher TNM staging (**Table** **1**). Kaplan–Meier analysis revealed that elevated expression of circ0006646 was related to worse overall survival in HCC cohort 2 (P = 0.0096) (Figure 1M). Besides, the overexpression of PTK2 was also correlated with poor prognosis, according to GEPIA database analysis (Figure 1N). Univariate and multivariate COX regression analyses indicated that overexpression of circ0006646 was an independent risk factor for HCC survival (HR = 8.902, P = 0.036; Figure 1O; Table S5, Supporting Information). Overall, we concluded that circ0006646 was prominently upregulated in HCC tissues and that higher circ0006646 expression might suggested a poor prognosis.

**Table 1:** Baseline information for clinical cohort 2.

	Variables	Expression		Total	p value
		Low	High		
Sex					1.000
	Male	17	35	52	
	Female	3	5	8	
Age[year]					0.855
	≤56	10	21	31	
	>56	10	19	29	
Grade					0.232
	I/II	16	26	42	
	III	4	14	18	
Tumor size					1.000
	≤6cm	15	30	45	
	>6cm	5	10	15	
T stage					0.073
	T1	17	25	42	
	T2 /T3	3	15	18	
TNM stage					0.050
	I	17	24	41	
	II‐VI	3	16	19	

### 2.3 Circ0006646 Enhances the Metastasis of HCC In Vitro and In Vivo

**Figure 2:**
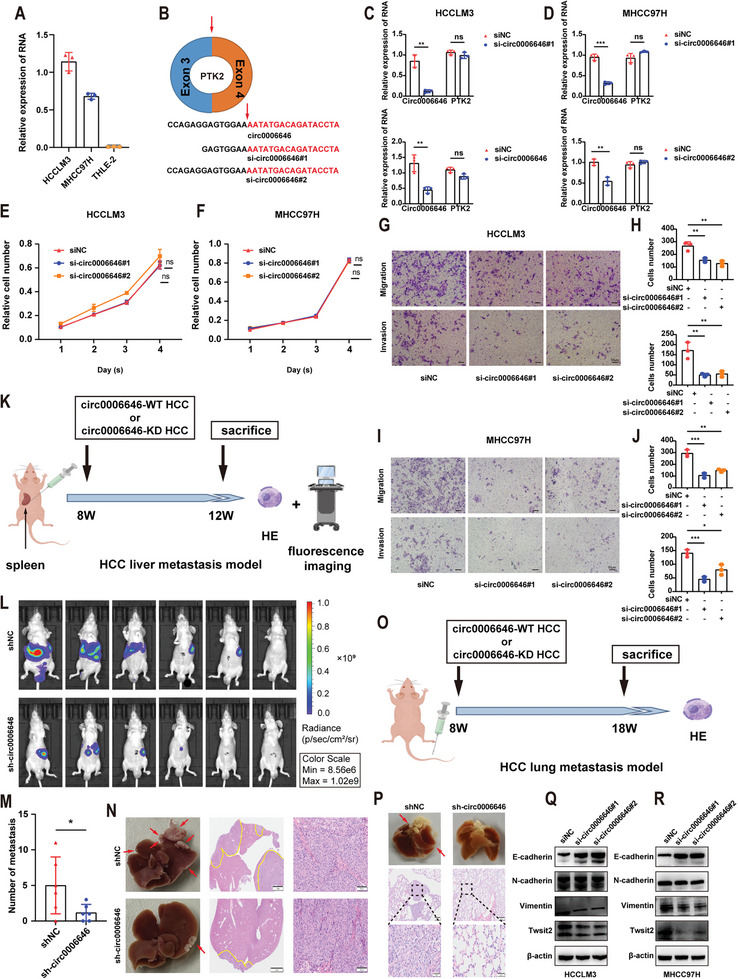
Circ0006646 promoted the metastasis ability of HCC. A) qRT‐PCR was used to measure the expression level of circ0006646 in HCC cell lines and a normal human liver cell. B) Two siRNAs were designed to target the junction site of Circ0006646. C,D) After cells were transfected with siNC and si‐circ0006646, the expression level of circ0006646 and PTK2 in HCCLM3 (C) and MHCC97H (D) were detected by qRT‐PCR. E,F) After cells were transfected with siNC and si‐circ0006646, the proliferation of HCCLM3 (E) and MHCC97H (F) were detected by CCK8 assay. G–J) After cells were transfected with siNC and si‐circ0006646, transwell assay was used to evaluate the migration and invasion ability of HCCLM3 (G,H) and MHCC97H (I,J). Scale bars, 50 µm. K–N) The spleens of mice were injected with luciferase‐labeled HCCLM3^circ0006646‐high^ or HCCLM3^circ0006646‐low^ to construct liver metastasis model, *n* = 6 mice/group (K). In vivo fluorescence imaging (L) was performed 4 weeks later. The number of liver metastases in two groups were compared (M). Representative photographs of liver showed the metastases (red arrows) and HE staining (N) was used to confirm the presence of metastases. Scale bars, 2 mm and 100 µm, respectively. O,P) The tail veins of mice were injected with HCCLM3^circ0006646‐high^ or HCCLM3^circ0006646‐low^ (O). The lung metastases were observed 10 weeks later. HE staining was used to confirm the presence of metastases (P). *n* = 5 mice/group. Scale bars, 200 and 50 µm, respectively. Q,R) After cells were transfected with siNC and si‐circ0006646, changes of markers of EMT in HCCLM3 (Q) and MHCC97H (R) were measured. Blots with antibodies recognizing the E cadherin, N cadherin, vimentin, Twist2 β‐Actin were shown. All experiments were performed in at least triplicate samples. Data were presented as the means ± SD. ^*^ *p* < 0.05, ^**^ *p* < 0.01, ^***^ *p* < 0.001. Student's *t*‐test was used for normally distributed data and Mann–Whitney U test was used for skewed distribution data.

To verify the effect of circ0006646 on the metastasis ability, we first measured circ0006646 expression levels in HCC cell lines and a human normal hepatocyte line (THLE‐2). A significant increase in circ0006646 expression was observed in HCC cell lines compared to normal cell line (THLE‐2) (**Figure** **2**A). We designed two siRNAs covering the back‐spliced site of circ0006646 (Figure 2B). The expression of circ0006646, but not PTK2, was silenced after transfection with two siRNAs, implying that the siRNAs precisely targeted circ0006646 instead of its parent genes (Figure 2C,D). Notably, circ0006646 did not interfere with the proliferation of HCC cells, as no significant difference was found in cell growth after silencing circ0006646 in a CCK8 assay (Figure 2E,F). Furthermore, HCCLM3 cells were transfected with sh‐circ0006646 and shNC lentivirus to form stable HCCLM3 with high circ0006646 expression (HCCLM3^circ0006646‐high^) and low circ0006646 expression (HCCLM3^circ0006646‐low^) for use in subsequent assays. There was no difference in the growth of HCCLM3^circ0006646‐high^ and HCCLM3^circ0006646‐low^ cells in vivo, an observation in line with the CCK8 assay results (Figure S3A–C, Supporting Information).

Transwell assays indicated that loss of circ0006646 restrained the migration and invasion abilities of HCCLM3 and MHCC97H cells (Figure 2G–J), which was also proven by wound‐healing assays (Figure S3D–G, Supporting Information). Conversely, overexpression of circ0006646 can promote HCC invasion and migration (Figure S3H–L, Supporting Information). Next, we investigated the role of circ0006646 in vivo utilizing the liver metastasis mouse model (Figure 2K). Luciferase‐labeled HCCLM3^circ0006646‐high^ and HCCLM3^circ0006646‐low^ cells were orthotopically injected into the spleens of mice. The metastatic ability of HCCLM3^circ0006646‐high^ cells in vivo was dramatically stronger according to the higher fluorescence intensity and more tumor metastases (Figure 2L,M). HE staining further validated that the area of metastatic nodules was larger in the HCCLM3^circ0006646‐high^ group (Figure 2N). These conclusions were in line with the results from a lung metastasis mouse model when cells were injected into the tail vein (Figure 2O,P).

In addition, epithelial‐to‐mesenchymal transition (EMT), which actuates the trans‐differentiation of epithelial cells to a mesenchymal phenotype, is one of the most classical changes involved in metastasis. In our research, when circ0006646 was silenced, the expression levels of E cadherin and vimentin, which are considered as the markers of EMT, increased and decreased, respectively (Figure 2Q,R). These changes were subsequently demonstrated by immunohistochemistry (IHC) staining and immunofluorescence (IF) assay (Figures S4A and S5A,B, Supporting Information). In conclusion, circ0006646 silencing inhibited HCC invasion and migration.

### 2.4 Circ0006646 Directly Interacts with the RNA‐Binding Protein NCL

**Figure 3:**
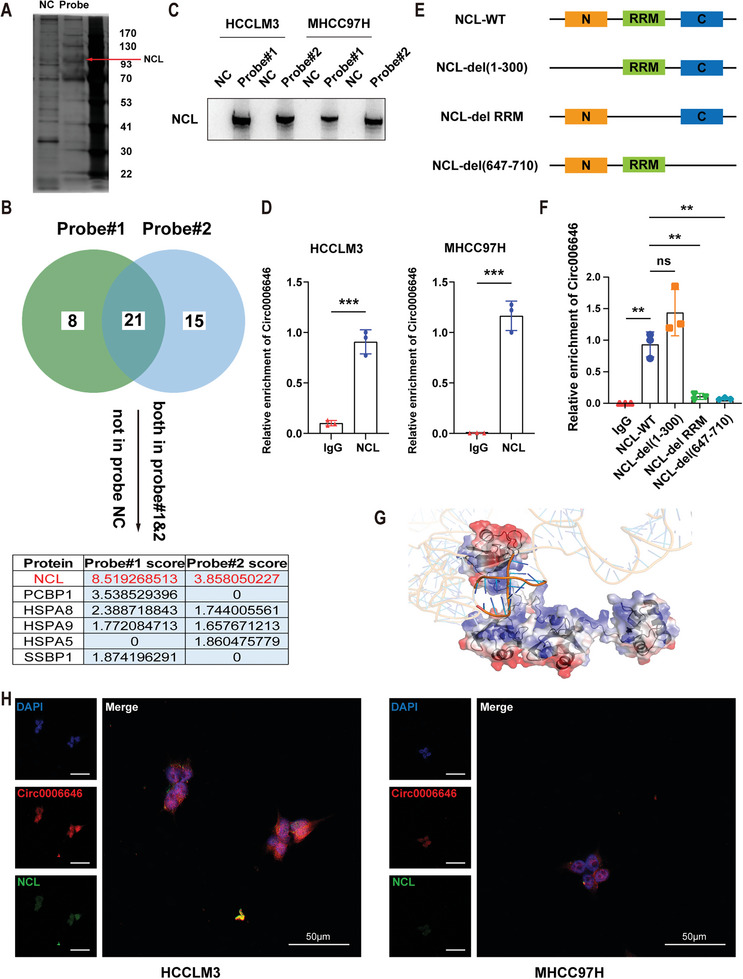
Circ0006646 combined with NCL in HCC cells. A) After cell lysis using RIPA, circ0006646 specific targeting probe and NC probe were used for pulldown assay, followed by protein silver staining. B) Selection strategies for potential binding proteins. After mass spectrometry, peptides which presented in two circ0006646 specific targeting probe groups but not in the NC probe group were considered as candidates for circ0006646 binding proteins. Select the one with the highest score for follow‐up verification. C) NCL enriched by two circ0006646 specific probes was verified. Blots with antibodies recognizing the NCL and β‐Actin were shown. D) The enrichment of circ0006646 in HCCLM and MHCC97H was detected by using NCL antibody and IgG antibody in RIP assay. E) Sketch‐map of three NCL truncated mutations. C: C‐terminal; N: n‐terminal; RRM: RNA recognition motif. F) Four different NCL plasmids (wild type and truncated mutation) were transfected into cells. In RIP assay, NCL antibody and IgG antibody were used to measure the enrichment of circ0006646. G) Graphical representation of a complex formed by the circ0006646 and NCL307‐647. The semi‐transparent blue–white–red surface depicts protein's contact potential (blue: positively charged, red: negatively charged). H) Representative IF images about the binding of circ0006646 to NCL were shown. DAPI staining represented the location of the nucleus. Red: circ0006646, green: NCL. All experiments were performed in at least triplicate samples. Data were presented as the means ± SD. ^**^ *p* < 0.01. Student's *t*‐test was used for normally distributed data and Mann–Whitney U test was used for skewed distribution data.

After demonstrating the potential therapeutic value of circ0006646, we wanted to understand how it works by exploring the specific molecular mechanisms. Numerous studies have demonstrated that circRNAs can bind, deliver, and isolate proteins to specific subcellular sites and function as stable scaffold molecules to regulate protein‒protein interactions. To explore the proteins interacting with circ0006646, two biotinylated probes specifically targeting it were used for pull‐down experiments. Subsequent protein silver staining results showed several instances of differential staining between 30 and 41 Kda, 41 and 70 Kda, 93 and 130 kDa (**Figure** **3**A). Next, among the numerous peptides revealed by mass spectrometry, NCL, which could be pulled down by both probes but was not present in the NC‐probe group, had the highest Sequest HT scores and was identified as a candidate protein interacting with circ0006646 (Figure 3B; Figure S6A–F, Supporting Information).

NCL is a highly enriched protein in nucleoli, and its abnormal expression and localization in cancer often affect the generation, proliferation and metastasis of tumor cells, leading to the progression of cancer.^[^ ^25^ ^]^ It was also verified that NCL can be markedly enriched by a circ0006646‐specific probe but not by an NC probe, in addition to a RIP assay that showed an obvious circ0006646 concentration by anti‐NCL (but not IgG) staining (Figure 3C,D).

To delineate the circ0006646‐binding site of the NCL protein, a series of flag‐tagged NCL truncation mutants were constructed and subsequently verified by WB assay (Figure 3E; Figure S7A, Supporting Information). Interestingly, after transfecting these mutant plasmids into cells and performing RIP experiments, we found that circ0006646 could not be enriched by anti‐FLAG antibodies when the RRM domain (307‐647) and C‐terminus (647‐710) were deleted, suggesting that these two domains may be responsible for the binding of NCL to circ0006646 (Figure 3F). The subsequent pulldown assay also proved this conclusion (Figure S7B, Supporting Information). Furthermore, we performed 3D structure modelling of circ0006646 using 3dRNA^[^ ^26^ ^]^ and got 3D structure of NCL307‐647, which is predicted by AlphaFold,^[^ ^27^ ^]^ from uniport (P19338, AF‐P19338‐F1). Then molecular docking was carried out on HDOCK webserver.^[^ ^28^ ^]^ We displayed the top‐ranked configuration according to score function defined by the docking software (Figure 3G). Circ0006646, whose backbone was negatively charged, attached onto a positively charged region rendered in blue of the protein (Figure S7C, Supporting Information). The co‐localization of circ0006646 and NCL in cells was also confirmed (Figure 3H). Therefore, NCL was the downstream binding protein of circ0006646.

### 2.5 Circ0006646 Blocks the Binding of Tripartite Motif‐Containing 21 (TRIM21) to NCL and Inhibits its Ubiquitination‐Mediated Degradation

**Figure 4:**
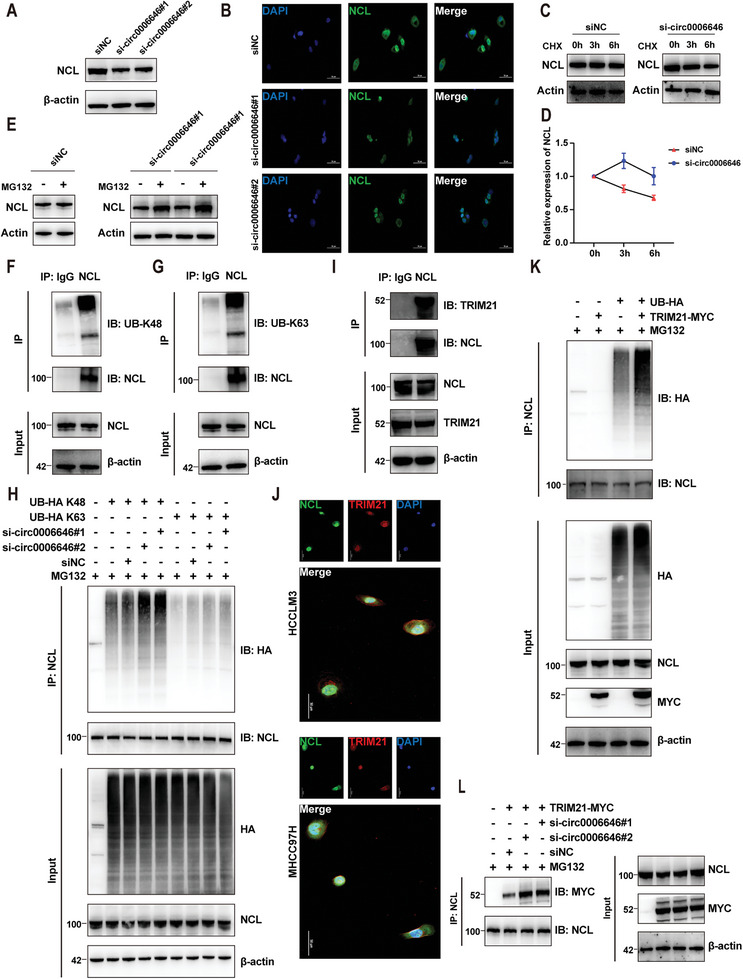
Circ0006646 inhibited the combination of TRIM21 and NCL to stabilize the expression of NCL. A) Cells were transfected with siNC and si‐circ0006646. Blots with antibodies recognizing the NCL and β‐Actin were shown. B) Representative IF images of NCL (green) were shown. DAPI staining represented the location of the nucleus. C) Cells were transfected with siNC and si‐circ0006646, and later treated with CHX for the indicated times. Blots with antibodies recognizing the NCL and β‐Actin were shown. D) Broken line graph represents the change of NCL expression at 0, 3, and 6 h after treatment of CHX. E) Cells were transfected with siNC and si‐circ0006646, and later treated with 10 µm MG132 for 6 h. Blots with antibodies recognizing the NCL and β‐Actin were shown. F,G) The HCCLM3 lysate was incubated with anti‐NCL and anti‐IgG, and later incubated with protein A/G magnetic beads. Blots with antibodies recognizing the UB‐K48 (F) or UB‐K63 (G) and NCL were shown. H) 293T cells transfected with HA‐tagged UB‐K48, HA‐tagged UB‐K63, siNC, and si‐circ0006646 were treated with 10 µm MG132 for 6 h. The 293T lysate was incubated with anti‐NCL, and later incubated with protein A/G magnetic beads. Blots with antibodies recognizing the HA and NCL were shown. I) The HCCLM3 lysate was incubated with anti‐NCL and anti‐IgG, and later incubated with protein A/G magnetic beads. Blots with antibodies recognizing the TRIM21 and NCL were shown. j) Representative IF images of NCL (green) and TRIM21 (red) were shown. DAPI staining represented the location of the nucleus. K) 293T cells transfected with HA‐tagged UB and MYC‐tagged TRIM21 were treated with 10 µm MG132 for 6 h. The 293T lysate was incubated with anti‐NCL, and later incubated with protein A/G magnetic beads. Blots with antibodies recognizing the HA and NCL were shown. L) 293T cells transfected with MYC‐tagged TRIM21, siNC, and si‐circ0006646 were treated with 10 µm MG132 for 6 h. The 293T lysate was incubated with anti‐NCL, and later incubated with protein A/G magnetic beads. Blots with antibodies recognizing the MYC and NCL were shown. All experiments were performed in at least triplicate samples. Data were presented as the means ± SD.

Knowing that NCL can bind to circ0006646, we further investigated how NCL was regulated to exert the downstream mechanism. We first focused on expression change of NCL. Surprisingly, circ0006646 silencing did not influence the transcription level of NCL but led to an attenuation of NCL protein expression, demonstrating that circ0006646 regulated NCL expression during the translation process (**Figure** **4**A; Figure S8A–C, Supporting Information). In parallel to the alteration in protein level, the results of IF assay also demonstrated the change in NCL expression (Figure 4B).

Furthermore, restraining intracellular protein synthesis with cycloheximide (CHX) did not reverse the reduction in NCL levels, while treatment with MG132, a specific proteasome inhibitor, restored NCL expression (Figure 4C–E; Figure S8D, Supporting Information). Therefore, we suspected that circ0006646 blocked the ubiquitination‐mediated degradation of NCL. To verify this conclusion, we first confirmed NCL ubiquitination in cells (K48‐linked and K63‐linked chains) (Figure 4F,G; Figure S9A, Supporting Information). Consistent with our hypothesis, when HA‐tagged ubiquitin (UB) plasmid (UB‐HA), Ub‐K48 plasmid (UB‐HA K48) and Ub‐K63 plasmid (UB‐HA K63) were transfected into cells, circ0006646 knockdown dramatically hindered the polyubiquitination of NCL, especially K48‐link ubiquitination (Figure 4H; Figure S9B, Supporting Information).

We then wondered whether the ubiquitination of NCL regulated by circ0006646 was mediated by E3 ubiquitin ligase. To test this conclusion, the NCL antibody was applied for a co‐IP experiment, and the obtained protein samples were silver stained followed by MS analysis (Figure S10A, Supporting Information). Among all the obtained peptides, TRIM21 was of great interest because it is an E3 ubiquitin ligase involved in tumor progression.^[^ ^29^ ^]^ Subsequently, co‐IP and IF experiments also confirmed the intracellular interaction between NCL and TRIM21 (Figure 4I,J; Figure S10B,C, Supporting Information). Next, co‐transfection of UB‐HA, MYC‐tagged TRIM21 (TRIM21‐MYC) plasmid prominently increased NCL ubiquitination, indicating that TRIM21 can indeed be regarded as an E3 ubiquitin ligase for NCL (Figure 4K). Further, co‐transfection of the TRIM21‐MYC plasmid and si‐circ0006646 enhanced the combination of NCL and TRIM21 (Figure 4L; Figure S10D, Supporting Information). Finally, the interaction of TRIM21 and the RRM domain (307‐647) or C‐terminus (647‐710) of NCL was also confirmed so that circ0006646 and TRIM21 might competitively combine with these two regions (Figure S10E, Supporting Information). Given this evidence, circ0006646 could stabilize NCL expression by preventing the integration of NCL into the E3 ligase TRIM21.

### 2.6 NCL Inhibits the Translation of TP53 by Combining its 5′ Untranslated Regions

**Figure 5:**
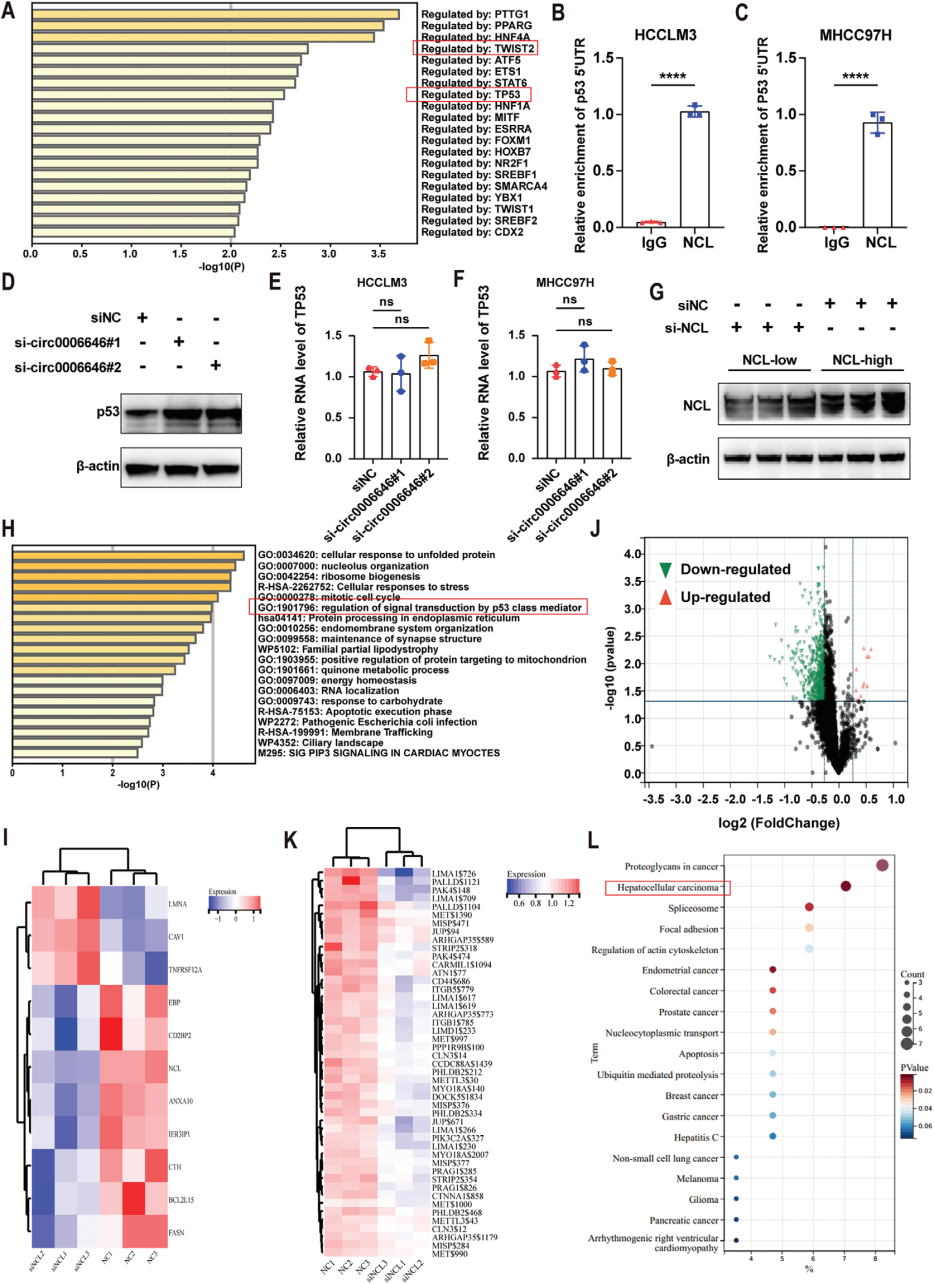
Multi‐omics revealed that NCL regulated the p53 protein level. A) Enrichment pathways of transcriptome sequencing in HCCLM3circ0006646‐high and HCCLM3circ0006646‐low. B,C) The enrichment of 5′UTR in RIP assay was detected by using NCL and IgG antibody in HCCLM3 (B) and MHCC97H (C). D) Cells were transfected with siNC and si‐circ0006646. Blots with antibodies recognizing the p53 and β‐Actin were shown. E,F) After cells were transfected with siNC and si‐circ0006646, the mRNA content of TP53 in HCCLM3 (E) and MHCC97H (F) was detected by qRT‐PCR. G) After cells were transfected with siNC and si‐NCL, change of NCL was measured. Blots with antibodies recognizing the NCL and β‐Actin were shown. H) Enrichment pathways of TMT‐based proteomics sequencing in HCCLM3^circ0006646‐high^ and HCCLM3^circ0006646‐low^. I) Heatmap of proteins associated with p53 pathway in proteomic sequencing. J) Heatmap of proteins associated with cell migration pathway in phosphoproteomics sequencing. K) Volcano plot of phosphoproteomics sequencing in HCCLM3^NCL‐high^ and HCCLM3^NCL‐low^. K) Enrichment pathways of phosphoproteomics sequencing in HCCLM3^NCL‐high^ and HCCLM3^NCL‐low^. All experiments were performed in at least triplicate samples. Data were presented as the means ± SD. ^****^ *p* < 0.0001. Student's *t*‐test was used for normally distributed data and Mann–Whitney U test was used for skewed distribution data.

When the regulatory mechanism of circ0006646 on NCL was elucidated, we attempted to analyze its downstream pathway. RNA sequencing was used to measure the transcriptome changes in HCCLM3^circ0006646‐high^ and HCCLM3^circ0006646‐low^. The p53 pathway was obviously enriched (**Figure** **5**A; Figure S11A, Supporting Information), as several studies have confirmed that NCL can bind to the 5′ untranslated region (UTR) of p53 mRNA, resulting in translational repression of p53.^[^ ^22^, ^30^ ^]^ Indeed, the binding of NCL to the 5′UTR of p53 mRNA was verified by RIP assay (Figure 5B,C), and the constant transcription level but significantly altered protein level of p53 also confirmed the dampening of p53 translation by NCL (Figure 5D–F). Interestingly, the Twist2‐related pathway, which has been reported by many studies to promote EMT^[^ ^31^, ^32^ ^]^ was also enriched and verified to reduce after circ0006646 knockdown (Figure 2Q,R).

In addition, the role of NCL in regulating protein phosphorylation has been less reported so that we performed the proteomics and phosphoproteomics of HCCLM3^NCL‐high^ and HCCLM3^NCL‐low^ (Figure 5G). At the protein level, proteomics sequencing showed that p53 pathway can still be enriched (Figure 5H,I). Meanwhile, the absence of NCL inhibited the phosphorylation of multiple proteins, suggesting NCL might influence the function of phosphokinase on target proteins (Figure 5J). “Cell migration” was the most enriched pathway in GO enrichment analysis of phosphoproteomics sequencing and some phosphorylation levels of migration‐related proteins were significantly altered (Figure 5J; Table S6, Supporting Information). It is worth noting that the phosphorylation level change of these proteins can significantly affect cancers, especially HCC (Figure 5K). To sum up, the change of NCL expression level significantly affects the p53 pathway in HCC.

### 2.7 Circ0006646 Regulates HCC Metastasis Through the NCL/p53/E Cadherin Axis

**Figure 6:**
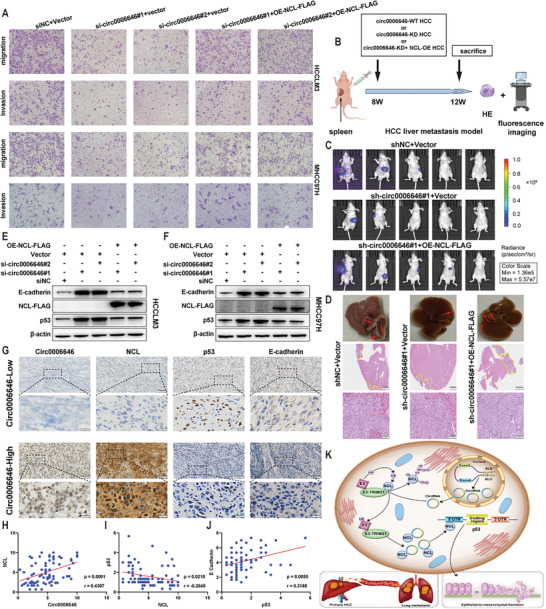
The role of circ0006646/NCL/p53/E cadherin axis in regulating HCC metastasis. A) HCCLM3 were transfected with siNC and si‐circ0006646, and later transfected with FLAG tagged OE‐NCL or vector. Transwell assay was used to evaluate the migration and invasion ability. B–D) HCCLM3 were transfected with shNC or sh‐circ0006646 lentivirus, and later transfected with FLAG tagged OE‐NCL lentivirus or vector. The spleens of mice were injected with these cells to construct liver metastasis model (B). In vivo fluorescence imaging was performed 4 weeks later, *n* = 5 mice/group (C). Representative photographs of liver showed the metastases (red arrows) and HE staining (D) was used to confirm the presence of metastases. Scale bars, 2 mm and 100 µm, respectively. E,F) HCCLM3 were transfected with siNC and si‐circ0006646, and later transfected with FLAG tagged OE‐NCL or vector. Expression changes of classical markers of EMT in HCCLM3 (E) and MHCC97H (F) were measured. Blots with antibodies recognizing the E cadherin, N cadherin, vimentin, and β‐Actin were shown. G) Representative ISH staining of circ0006646 and IHC staining of NCL, p53, and E cadherin were shown. Scale bars, 100 and 20 µm, respectively. H–J) The expression correlations between circ0006646 and NCL (H), NCL and p53 (I) and p53 and E cadherin (J) were evaluated. K) Schematic diagram of circ0006646 regulating HCC metastasis. All experiments were performed in at least triplicate samples. Data were presented as the means ± SD. Student's *t*‐test was used for normally distributed data and Mann–Whitney U test was used for skewed distribution data.

Restoration of NCL protein levels through transfection of the Flag‐labeled NCL overexpression plasmid rescued the impaired HCC metastasis capacity caused by circ0006646 depletion in vivo and in vitro (**Figure** **6**A–D; Figure S12A–F, Supporting Information). Besides, the elevated expression levels of p53 and E cadherin caused by circ0006646 silencing were also restored by the overexpression of NCL (Figure 6E,F; Figure S12G–J, Supporting Information). Finally, to verify the universality of the circ0006646/NCL/p53/E cadherin axis, we further chose HCC patients (cohort 1) with distinct circ0006646 expression levels to evaluate the relevance of this axis. In line with previous results, IHC data indicated the clinical correlation of this axis (Figure 6G–J; Figure S13A–C, Supporting Information). These results fully demonstrated the role of circ0006646/NCL/p53/E cadherin axis in the regulation of HCC metastasis (Figure 6K).

### 2.8 Circ0006646 is a Potential Therapeutic Target for HCC Patients

**Figure 7:**
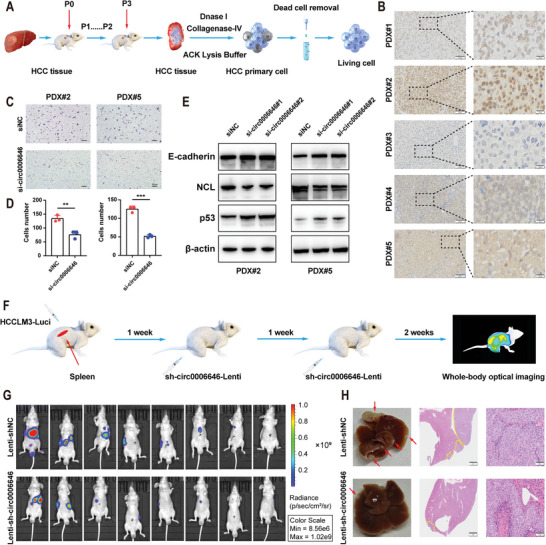
Circ0006646 might be a potential target for the treatment of HCC metastasis. A) The extraction process of primary HCC cells was presented. After 3 successive generations of HCC patient tumor tissue were cultured in mice, nucleic acid, erythrocyte, and extracellular matrix were digested with deoxyribonuclease, ACK lysing buffer, and collagenase‐IV, respectively. Then, the dead cells were removed, and the living primary HCC cells were obtained. B) ISH staining of circ0006646 was performed on 5 PDX models. Scale bars, 100 and 20 µm, respectively. C,D) After 2 primary HCC cells were transfected with siNC and si‐circ0006646, transwell assay was used to evaluate the migration and invasion ability. Scale bars, 50 µm. E) Two primary HCC cells were transfected with siNC and si‐circ000664. Blots with antibodies recognizing the E cadherin, NCL, p53, and β‐Actin were shown. F) Schematic diagram of simulated systemic therapy for HCC patients. The spleens of mice were injected with lucifera‐labeled HCCLM3 to construct liver metastasis model in vivo. shNC or sh‐circ0006646 lentivirus was injected through tail veins at the second and third weeks, followed by in vivo fluorescence imaging at the fourth week. G) Liver metastases were evaluated by in vivo fluorescence imaging, *n* = 8 mice/group. H) Representative photographs of liver showed the metastases (red arrows). HE staining was used to confirm the presence of metastases. Scale bars, 2 mm and 100 µm, respectively. All experiments were performed in at least triplicate samples. Data were presented as the means ± SD. ^*^ *p* < 0.05, ^**^ *p* < 0.01, ^***^ *p* < 0.001. Student's *t*‐test was used for normally distributed data and Mann–Whitney U test was used for skewed distribution data.

Finally, we wondered whether this phenomenon would also be applicable to HCC patients. First, we constructed an HCC PDX model (**Figure** **7**A). After growing in mice for three consecutive generations, the tumor tissue was harvested, and deoxyribonuclease, ACK lysis buffer, and collagenase‐IV were added to remove nucleic acids, red cells, and extracellular matrix proteins, respectively. Next, a magnetic column was used to remove the dead cells so that living primary HCC cells were obtained. Subsequently, we performed IHC staining with circ0006646‐probe on PDX models of 5 patients and selected the two PDXs (2 and 5) with the highest relative expression levels of circ0006646 to extract primary tumor cells (Figure 7B). Through the transwell assay, we found that circ0006646 silencing could also inhibit the metastatic ability of primary HCC cells (Figure 7C,D). In addition, the protein level alternation of NCL, p53, and E cadherin were consistent with the results in HCC cell lines (Figure 7E).

After confirming circ0006646 was a potential therapeutic target for HCC patients by applying the PDX model, we imitated clinical systemic therapy by injecting sh‐circ0006646 or shNC lentivirus (two injections, one week apart) into the tail vein of mice, whose spleens were injected with luciferase‐labeled HCCLM3 cells (Figure 7F). Injection of sh‐circ0006646 lentivirus was shown to reduce the metastatic ability of HCC cells in vivo, as measured by lower fluorescence intensity, fewer metastases, and smaller tumor area (Figure 7G,H; Figure S14A,B, Supporting Information). Overall, circ0006646 was a therapeutic target with clinical translational value.

## 3 Discussion

Metastasis, which is one of the major obstacles in HCC therapy, accounts for the majority of cancer‐related mortality.^[^ ^3^, ^33^, ^34^ ^]^ Numerous HCC patients are diagnosed at advanced stages and are amenable to only systemic therapies rather than surgery. Despite the remarkable advances of systemic therapies, only a small percentage of patients experience long‐term clinical benefits.^[^ ^35^ ^]^ Even sorafenib, which was once the only therapeutic strategy for advanced HCC patients, has been verified to expedite metastasis rather than block it. Extensive sequencing methods have suggested that genetic mutations alone are inadequate to explain the events observed during metastasis, indicating that additional properties support successful metastasis. Currently, epigenetic regulation is considered a powerful contributor.^[^ ^37^ ^]^ Metastasis, as a multistep event, seems more reasonably regulated by dynamic epigenetics than static gene mutations.

CircRNAs, as emerging epigenetic modulators, are attracting increasing attention due to their extensive role in tumors. With the development of bioinformatics and high‐throughput sequencing techniques, numerous studies have revealed their tissue‐specific and cell‐specific roles, as well as their molecular mechanisms in HCC proliferation, apoptosis, and drug resistance.^[^ ^38^, ^39^ ^]^ It has been reported that circRNAs regulate metastasis by influencing EMT, angiogenesis, and resistance to targeted treatment agents.^[^ ^40^, ^41^ ^]^ However, the current understanding of the mechanism of circRNAs in HCC metastasis is not comprehensive. In view of this, our study revealed that circ0006646, which has never been studied, could affect the EMT process by blocking the binding of E3 ligase to NCL. In addition, we also revealed that circ0006646 mediated the phosphorylation level of HCC cells by stabilizing the expression level of NCL.

The localization of circRNAs greatly affects their function. When located in the nucleus, they are mainly involved in transcriptional regulation or nucleolar formation, while cytoplasmic circRNAs mainly act as microRNA (miRNA) sponges or interact with proteins. In the past few years, many studies have revealed that circRNAs exerted biological functions by acting as competing endogenous RNA (ceRNA). However, due to the difficulty of individual transcript expression to reach the level required for highly expressed miRNAs and the limitation of experimental verification, the ceRNA hypothesis has been controversial.^[^ ^42^ ^]^ The interaction of circRNAs with proteins in HCC is attracting increasing attention. Through binding with proteins, circRNAs can regulate synthesis and degradation or translocate proteins to particular subcellular fractions, thereby affecting the progression of HCC. For instance, Shi et al. reported that the tumor‐suppressive function of circPABPC1 was achieved by linking ITGB1 to the 26S proteasome for degradation.^[^ ^43^ ^]^ We found that circ0006646 was located in both the nucleus and cytoplasm, and a series of specific peptides were identified as circ0006646 interactors by RNA pulldown, RIP, and MS assays. Among these interactors, NCL was a classical RNA‐binding protein with the highest score.

The structure of NCL is complex and varied, containing an intrinsically disordered N‐terminal region, followed by four RNA‐binding domains (RBDs) and an arginine–glycine–glycine‐rich (RGG) domain at the C‐terminus.^[^ ^44^ ^]^ Due to the existence of the RGG domain, NCL has been proven to preferentially bind G‐quadruplex (G4) structures, which are G‐quartets formed by self‐recognition of guanines, to act on diverse processes such as telomere biology, polyadenylation, genome instability, transcription termination, and miRNA production.^[^ ^19^, ^45^ ^]^ Our experiments revealed the conformation of circ0006646 bound with RBD. Incidentally, it is worth noting that RBD was made up of several small rigid domains linked by relatively flexible single‐file peptide, and the residues of and in the vicinity of the linker were mainly positively charged, which was attracted to the negatively charged RNA. At the experimental level, we also found that after truncating the RBD and C‐terminus of NCL, its ability to bind circ0006646 was greatly weakened.

Multiple functions of NCL influence the cellular processes of oncogenesis, including ribosome biogenesis, genomic instability, angiogenesis, and lymphangiogenesis.^[^ ^44^ ^]^ To some extent, NCL functions require PTM, such as phosphorylation, glycosylation, and methylation. Liang et al. reported that PTENβ restrained the transcription of rDNA through dephosphorylation of NCL at Thr84.^[^ ^46^ ^]^ In addition, the RGG domain of NCL is the primary substrate for methylation, and the Rio domain‐containing protein RioK1 can act as an adapter protein by recruiting NCL to the protein arginine methyltransferase 5 complex, thereby driving its methylation.^[^ ^47^ ^]^ However, ubiquitination of NCL has rarely been reported. Our study showed that the protein level of NCL decreased after circ0006646 silencing, and this change was not prevented by CHX but could be rescued by MG132. Subsequently, it was demonstrated that the absence of circ0006646 accelerated the protease‐mediated degradation of NCL triggered by ubiquitination. Distinct polyubiquitination linkages mediate disparate protein fates. For example, K63‐linked polyubiquitination is involved in signal transduction, while K48‐linked polyubiquitination stimulates proteasome‐mediated degradation.^[^ ^48^, ^49^ ^]^ Consistent with this conclusion, the stabilizing effect of circ0006646 on NCL protein levels was accomplished by blocking K48‐linked polyubiquitination.

E3 ligases account for the diverse ubiquitination patterns and we confirmed that TRIM21 is an E3 ligase of NCL.^[^ ^50^ ^]^ TRIM21, characterized by a RING domain for the E3 ubiquitin ligase, is a member of the TRIM family and plays a key role in the origin and development of cancers.^[^ ^29^ ^]^ Subsequent mechanistic studies confirmed that circ0006646 could prevent TRIM21 from anchoring ubiquitin to NCL. Based on previous 3D structural predictions of the interaction of circ0006646 and NCL, we speculated that this combination may change the structure of NCL, leading to a distinct conformation that was not able to be recognized by TRIM21. To our knowledge, TRIM21 has not been previously reported as an E3 ligase of NCL.

Subsequently, we attempted to explore the downstream changes in the NCL pathway by combined utilization of transcriptome and protein sequencing. Among all the altered pathways, the enrichment of p53 pathway caught our attention, as it was enriched in both transcriptomic and proteomic sequencing. Takagi et al. proved that NCL suppressed p53 translation by attaching to the 5′ UTR of p53 mRNA,^[^ ^22^ ^]^ which was also verified by our research. Moreover, p53 can bind to the enhancer regions of the CDH1 (encodes E‐Cadherin) enhancer, which is critical for sustaining CDH1 expression.^[^ ^51^, ^52^ ^]^ Nevertheless, although CDH2 (encodes N‐Cadherin) also contains various theoretical sites for interaction with p53, none of them are the enhancer regions.^[^ ^51^ ^]^ This conclusion might support the phenomenon that E cadherin was the most significantly changed EMT marker in our study. Correlation analysis of gene and protein expression in clinical samples also indicated the potential regulatory mechanism of the circ0006646/NCL/p53/E cadherin axis and its nonnegligible role in HCC metastasis.

Translating molecular knowledge into precision oncology and even promoting the implementation of molecular‐based randomized controlled trials is the current development trend to improve HCC therapy. Recently, the evolution of preclinical models with biological and genetic characteristics of human cancers, such as PDX, has initiated a new era for more efficacious translation of molecular research into novel treatment.^[^ ^53^, ^54^, ^55^ ^]^ Therefore, we constructed PDX models of HCC to verify the possibility of circ0006646 as a clinical therapeutic target. Indeed, the preliminary experimental results revealed that circ0006646 silencing influenced the circ0006646/NCL/p53/E cadherin axis in PDX models. Finally, we simulated systemic therapy by injecting a circ0006646 knockdown lentivirus into the mice and substantiated the inhibitory effect of this treatment on metastasis.

In summary, this study described the integrated multi‐omics landscape of circRNA‐mediated NCL ubiquitination degradation in HCC metastasis. Circ0006646, a novel up‐regulated RNA in HCC, could promote metastasis by inhibiting K48 ubiquitination to stable NCL expression. P53 pathway played an important role in this biological process to mediate the occurrence of EMT. In addition, how to use a novel gene delivery system to interfere with the expression of circ0006646 more efficiently and how NCL regulates HCC metastasis through phosphorylation still need to be further explored.

## 4 Experimental Section

### Patient Cohorts and Tumor Tissues

The tumor tissues were collected from the surgical samples of 76 HCC patients (HCC cohort 1) from Affiliated Hangzhou First People's Hospital, Zhejiang University School of Medicine from 2020 to 2022. Frozen tissues were used for quantitative reverse transcription polymerase chain reaction (qRT‐PCR) and western blotting (WB) analysis, while formaldehyde‐fixed and paraffin‐embedded tissues were used for in situ hybridization (ISH) experiments. 60 HCC tissue cDNA microarrays (HCC cohort 2) with clinical information were obtained from Shanghai Outdo Biotech Company, China. Ethical approval for this study was provided by the Ethical Committee of Affiliated Hangzhou First People's Hospital, Zhejiang University School of Medicine and Shanghai Outdo Biotech Company, China. All study was conducted in accordance with both the Declarations of Helsinki and Istanbul. Written informed consent was provided by all patients.

### Cell Culture, Transfection, Lentivirus Infection, and Function Experiment

All plasmids were constructed by RepoBio (Hangzhou, China) and details are provided in Supporting Information.

### Quantitative Reverse Transcription Polymerase Chain Reaction (qRT‐PCR), Western Blotting (WB) and Coimmunoprecipitation Assay (Co‐IP)

Details are provided in Supporting Information. Primer sequence is shown in Table S1 (Supporting Information). The antibodies used in the study are listed in Table S2 (Supporting Information).

### RNA Immunoprecipitation (RIP), Pull Down Assay, Fluorescence In Situ Hybridization (FISH), In Situ Hybridization (ISH) and Immunofluorescence (IF)

Details are provided in Supporting Information. Sequences for the nucleic acids used in the study are listed in Table S3 (Supporting Information).

### Animal Experiments

Details are provided in Supporting Information.

### Transcriptome and circRNA Sequencing

Details are provided in Supporting Information.

### PDX Model

PDX models were established by tailoring the HCC tissues of patients and inoculating them into immunodeficient mice. The detailed process can be found in our previous research.^[^ ^56^ ^]^

### Tandem Mass Tags (TMT) ‐Based Phosphoproteomics

Details are provided in Supporting Information.

### Statistical Analysis

Continuous variables were reported as means ± standard deviation (SD) or median. If the data was normally distributed, Student's *t‐*test was used, otherwise Mann–Whitney U test was used. Categorical variables were presented as numbers and percentages and were compared by the Chisquare test. Kaplan–Meier method and log‐rank test were applied to analyze the survival curves. Independent prognostic factors were predicted by the cox proportional hazards model. SPSS V.21.0 software (IBM, NY) and GraphPad Prism 9 were used for statistical analysis.

## Conflict of Interest

The authors declare no conflict of interest.

## Supporting information

Supporting Information

## Data Availability

The data that support the findings of this study are available from the corresponding author upon reasonable request.
