# Supplementary material for: Integrated Multiomics Reveals Silencing of has_circ_0006646 Promotes TRIM21‐Mediated NCL Ubiquitination to Inhibit Hepatocellular Carcinoma Metastasis
Source: Adv Sci (Weinh). 2024 Feb 15;11(16):2306915. doi: 10.1002/advs.202306915 (PMC11040345; doi:10.1002/advs.202306915)
Supplement: Supplementary file 1 — Supporting Information [file ADVS-11-2306915-s001.pdf]

ADVANCED SCIENCE
Open Access

Supporting Information

for *Adv. Sci.*, DOI 10.1002/advs.202306915

Integrated Multiomics Reveals Silencing of has_circ_0006646 Promotes TRIM21-Mediated NCL Ubiquitination to Inhibit Hepatocellular Carcinoma Metastasis

Xin Hu, Guanrong Chen, Yingchen Huang, Qiyang Cheng, Jianyong Zhuo, Renyi Su, Chiyu He, Yichao Wu, Zhikun Liu, Beng Yang, Shuai Wang, Lijun Meng, Shusen Zheng, Di Lu, Qiang Wei, Jiayin Yang, Xuyong Wei, Ronggao Chen and Xiao Xu**

Integrated multiomics reveals silencing of has_circ_0006646 promotes TRIM21-mediated NCL ubiquitination to inhibit hepatocellular carcinoma metastasis

Xin Hu, Guanrong Chen, Yingchen Huang, Qiyang Cheng, Jianyong Zhuo, Renyi Su, Chiyu He, Yichao Wu, Zhikun Liu, Beng Yang, Shuai Wang, Lijun Meng, Shusen Zheng, Di Lu, Qiang Wei, Jiayin Yang, Xuyong Wei, Ronggao Chen, and Xiao Xu

1. Supplementary method
2. Supplementary figure
3. Supplementary table 1. Primer sets used for qRT-PCR
4. Supplementary table 2. Antibodies used in the study
5. Supplementary table 3. Sequences for the nucleic acids
6. Supplementary table 4. The differential expression circRNAs
7. Supplementary table 5. Univariate and Multivariate
8. Supplementary table 6. Top 10 terms in GO Enrichment Analysis of TMT-based phosphoproteomics sequencing

Cell culture, transfection and function experiment

HEK293T cell lines were purchased from American Type Culture Collection (ATCC), USA. Normal human liver cell (THLE-2) was purchased from Chinese Tissue Culture Collections, China. Human HCC cell lines (HCCLM3 and MHCC97H) were purchased from Shanghai Institute of Cell Biology, Chinese Academy of Sciences.

HEK293T, HCCLM3 and MHCC97H cell lines were cultured in Dulbecco's modified Eagle's medium (DMEM) (BI, Israel), which was composed of 10% fetal bovine serum (FBS) (BI, Israel) and 1% penicillin-streptomycin (Gibco, USA). The cells were maintained at 37°C in a humidified incubator with 5% CO2

THLE-2 cell was maintained in Bronchial Epithelial Cell Growth Medium (Lonza, Switzerland) with 5 ng/mL EGF and 10% FBS. The petri dishes were coated with Bronchial Epithelial Cell Basal Medium (Lonza, Switzerland), fibronectin (Sigma, Germany), bovine collagen type I (Sigma, Germany) and bovine serum albumin (Sigma, Germany) 24h in advance.

HCCLM3-Luci cells were constructed with HCCLM3 infected by LV-Luc2-ZsGreen-Puro lentivirus Stably expression clone was screened with DMEM containing puromycin.

UB-HA, UBK48-HA, UBK63-HA plasmid were constructed by pcDNA3.1-HA vector, TRIM21-MYC was constructed by pcDNA3.1-MYC vector and NCL-FLAG was constructed by pcDNA3.1-FLAG-GFP vector. Sequencing was used to confirm all constructs. SiRNA and plasmids were transiently transfected into cells by means of DNA transfection reagent (Polyplus, France) based on the manufacturer's instructions.

For CCK8 assay, a 96-well plate was inoculated with cells and cultured for 24 hours, 48 h, 72 h, and 96 h. Removing culture medium and add 10 μl of CCK-8 solution (MCE, China) to each

well and culture at 37°C for 1 hour. Then spectrometric absorbance was measured at 450nm with the microplate reader (SpectraMax iD5; MD, USA).

For transwell assay, cells were suspended in 100 mL of FBS-free DMEM medium and then introduced into a transwell filter's (Corning, USA) upper compartment for migration test, or cells were inoculated into the upper cavity coated with matrix glue for invasion test. The lower cavity was supplemented with 800μl of DMEM medium that contained 10%FBS. After 48h culture, cells were stained with Fast Wright's-Giemsa Stain (Yeasen Biotechnology, China) at room temperature. Finally, the images were captured by an inverted microscope (CKX53, Olympus, Japan).

For wound healing assay, cells were inoculated into 12-well plates. After cell attachment, they were mechanically destroyed with a sterile 200 μl of pipette tip to form a linear wound. Finally, the images were captured by an inverted microscope and the cellular migration zone was computed by image J software after 0h and 48h.

RT-PCR, WB and coimmunoprecipitation assay (Co-IP)

After treatment, cells were gathered and the total RNA was extracted utilizing the Trizol reagent (Sigma, Germany). The purity and concentration of RNA was measured by NanoDrop 2000 (ThermoFisher Scientific, USA). The RNA in each sample was reversed-transcribed into cDNA using the HiScript II Q RT SuperMix (Vazyme, China) kit. qRT-PCR was then performed using the SYBR Green Master Mix kit and the QuantStudio system (ABI, USA). Expression levels of the obtained target genes have been standardized to GAPDH or β-Actin expression levels.

Cells were collected and cleaved using RIPA lysis buffer (Millipore, USA) at 4°C for 30

minutes. After measuring the protein content using the BCA kit (ThermoFisher Scientific, USA), 5 x loading buffer were added, and the resulting mixture was subjected to incubation at 95°C for a duration of 10 minutes. The isolation of total proteins was accomplished through the utilization of SDA-PAGE gel electrophoresis, followed by their subsequent transfer onto a PVDF membrane (Millipore, USA). The primary antibody was incubated with the membranes at 4°C for the duration overnight. and washed with TBST buffer (Fudebio, China) three times for 15 minutes. Subsequently, the membranes were subjected to a 2-hour incubation with secondary antibodies at room temperature. The ECL chemiluminescence detection kit (Fudebio, China) was used to detect relevant proteins level.

Co-IP was completed using Classic Magnetic IP/Co-IP Kit (ThermoFisher Scientific, USA). 1 × 107 cells were lysed with 500 μL of cell lysis solution and incubated with 6 μg of IP antibody at 4 °C with rotation overnight to form immune complexes. 30μL of Pierce Protein A/G Magnetic Beads were mixed with the immune complexes and incubated at room temperature with rotation for 1 hour. Then, magnetic beads were washed 3 times in magnetic separator with cold cell lysis solution, followed by elution with 80 μL of cell lysis solution. After adding 5 x loading buffer, the mixture was incubated at 95°C for 10 minutes.

Lentivirus infection

Lentivirus-shRNA-circ0006646 sequence was cloned into pLKO.1-Puro plasmid vector. 293 T cells were transfected with shRNA plasmid vectors and packaged plasmid vectors and the culture medium was replaced 24 h later. After 72 hours, virus particles were collected from the

supernatant, then the virus was purified by centrifugal. Cells were infected with lentivirus (MOI=10) and polybrene (2 μg/mL) for 48 h and screened with puromycin.

RNA Immunoprecipitation (RIP)

The Magna RIP RNA-Binding Protein Immunoprecipitation Kit (Millipore, USA) was utilized to conduct RIP experiments. 1×107 cells were cleaved with RIP lysis buffer and stored at -80°C. At room temperature, the specific antibodies and magnetic beads were incubated with rotation for 60 minutes. At the same time, cell lysate underwent a thawing process followed by centrifugation at 12000 rpm and 4°C for a duration of 10 minutes. The supernatant after centrifugation was gathered and incubated overnight at 4°C with magnetic beads coated with antibodies. On the second day, magnetic beads were washed 5 times with RIP wash buffer, then eluted and purified RNA was prepared.

Pull down assay

The pulldown experiment was carried out with Pierce Magnetic RNA-Protein Pull-Down Kit (ThermoFisher Scientific, USA). 1×107 cells were lysed with the cell lysis buffer containing protease inhibitors and RNA enzyme inhibitors. Biotinylated RNA probes and control probes were incubation with streptavidin-coated magnetic beads under rotational agitation at room temperature for 60 minutes. Next, streptavidin magnetic beads fixed with probes were incubated with the cell lysates above for 2 hours with rotation at 4°C. Finally, the magnetic beads were cleaned 3 times, and then the protein on the beads was eluted.

Fluorescence in situ hybridization (FISH)

Cells were fixed with 4% paraformaldehyde for 10min and then incubated with immunostaining permeate (Beyotime, China) for 10min at room temperature. Then, cells were incubated with Cy3-labeled RNA probes overnight at 37°C away from light, and washed with 4×SSC (Solarbio, China) 3 times, 2×SSC once, and 1×SSC once. Finally, the nucleus was stained with DAPI and laser confocal microscope (Olympus FV3000, Japan) was used to obtain fluorescence images.

In situ hybridization (ISH)

After organization fixed and dehydration, the sections were digested with proteinase K (20ug/ml) working solution. After further treatment with prehybridization and hybridization solution, sections were incubated overnight with hybridization solution containing probe at 40°C. Next, the sections incubated overnight with anti-digoxin-HRP antibody at 4°C. Finally, sections were stained with DAB and nuclei with hematoxylin staining.

Immunofluorescence (IF)

About 2000 cells were inoculated on Nun cell culture dishes (ThermoFisher Scientific, USA) and cultured at 37°C overnight. On the second day, the cells were fixed with 4% fromaldehyde for 10 mins, incubated with immunostaining permeate (Beyotime, China) for 10min, and blocked with 10% BSA in PBS for 1hour. Subsequently, the cells were subjected to an overnight incubation at a temperature of 4°C in the presence of a specific primary antibody. Subsequently, on the third day, the cells were subjected to incubation with fluorescent secondary antibodies (Alexa Fluor Plus, ThermoFisher Scientific, USA) for 2 hours at room temperature. The nucleus

was stained with DAPI and laser confocal microscope (Olympus FV3000, Japan) was used to obtain fluorescence images.

Molecular docking

Before using HDOCK[1] to predict binding complex, we firstly used 3dRNA webserver[2] to generate 3D structure of RNA in pdb format. In the webserver, select "circular RNA" for RNA type, and "Example1" for some default setting. Paste our RNA sequence into the Sequence box. Click "yes" for Predict 2nd structure, and select "RNAfold" for 2nd Structure Prediction Method. Then submitted the job to wait the prediction result. As for the human NCL307-647 structure, we firstly got the entire human NCL structure, which is predicted by AlphaFold[3], from uniprot webserver (uniprot number is P19338). Then, the residues ranging from 307 to 647 are separated to a new pdb file.

Once getting pdb structures of RNA and NCL307-647, we refer to HDOCK webserver to predict complex. The process is straightforward. Upload the protein pdb file as receptor, and the RNA pdb file as ligand. Then, submit the prediction job. As demonstrated in HDOCK paper[4]: "With the structures modeled by the server or uploaded by users, the workflow now enters the last step, traditional global docking. Here, HDOCKlite, a hierarchical FFT-based docking program developed in our group, is used to globally sample putative binding orientations". A database (i.e., PDB database) is needed for template-based docking if a complex template is found through sequence similarity search. In ref[4], the author stated: "For the sake of computational efficiency and least interruption of service, a local copy of the PDB database is maintained on the HDOCK web server and updated monthly". As webserver users, we do not

need to select any kind of database for the prediction.

Mouse xenograft experiment

Male BALB/c mice were purchased from the Zhejiang Experimental Animal Center and raised in specific pathogen-free environments under 12-hour light-dark cycles, with adequate water and food. The animal experiments were approved by the animal ethics committee of The First Affiliated Hospital, Zhejiang University School of Medicine. Experiments were performed in strict accordance with the NIH Guide for the Care and Use of Laboratory Animals.

The effect of circ0006646 silencing on HCC proliferation was studied using male BALB/c nude mice (4-5 weeks). A total of 10 BALB/c nude mice were randomly allocated into two groups. One group was injected subcutaneously with 5×106 circ0006646 stabilized-knockdown HCCLM3 cells (HCCLM3circ0006646-low). The other group of mice were injected with 5×106 HCCLM3 control cells (HCCLM3circ0006646-high). After 4 weeks, all the mice were sacrificed, the xenografts were removed, the length and width of the tumors were measured with calipers, and the weight of the tumors were weighed.

Lung metastasis model: HCC lung metastasis was performed with male BALB/c nude mice (4-5 weeks). 20 BALB/c nude mice were randomly allocated into two groups. One group was injected with 3×106 circ0006646 stabilized-knockdown HCCLM3 cells (HCCLM3circ0006646-low) from the tail vein. The other group was injected with 3×106 HCCLM3 control cells (HCCLM3circ0006646-high). 10 weeks later, mice were sacrificed and their lungs were extracted for subsequent analysis.

Liver metastasis model: HCC liver metastasis was conducted using male BALB/c nude mice (4-5 weeks). 20 BALB/c nude mice were randomly allocated into two groups. One group was

injected with 3×106 circ0006646 stabilized-knockdown luciferase-labeled HCCLM3 cells from the spleen. The other group was injected with 3×106 HCCLM3 control cells. After 4 weeks, mice were injected intraperitoneally with fluorescein sodium salt (Sigma, Germany) for fluorescence imaging. Finally, all mice were sacrificed and their livers were removed for subsequent study.

Transcriptome sequencing

Total RNA was extracted from each sample using Trizol. Quality of RNA was evaluated through the utilization of a NanoDrop spectrophotometer (ThermoFisher Scientific, USA) to ensure high quality RNA was used for sequencing. Using the TruSeq RNA Library Prep Kit (Illumina, USA) to prepare RNA libraries. Poly(A) mRNA was enriched and fragmented, followed by cDNA synthesis, end repair, and adapter ligation. Library quality was assessed using a Qubit fluorometer and an Agilent Bioanalyzer. Library concentration was adjusted to ensure equal representation of each sample. RNA libraries were sequenced on a next-generation sequencing platform NovaSeq. Paired-end sequencing was typically used to generate high-quality reads. Raw sequencing data was processed using quality control software to remove low-quality reads and adapter sequences. Reads were then aligned to a reference genome or transcriptome using software HISAT2. Gene expression levels were quantified using software HTSeq. EdgeR software was employed to detect genes exhibiting differential expression across the samples. Functional analysis was performed using software such as GOSeq or KEGG to identify enriched biological pathways and gene ontology terms. The above experimental section was completed by Hangzhou Cosmos Wisdom.

CircRNA sequencing

The RNA Nano 6000 Assay Kit of the Bioanalyzer 2100 system (Agilent Technologies, CA, USA) was utilized to evaluate the quantity and quality of RNA. A total amount of 2μg RNA per sample was applied as input material for the preparations of the RNA samples. Following the qualification of the library, the various libraries were being pooled based on their effective concentration and the desired quantity of machine-generated data, and subsequently subjected to sequencing using the Illumina NovaSeq 6000 platform. Raw data in fastq format underwent processing via in-house perl scripts. The reference genome and gene model annotation files were procured from the genome website in a direct manner. The circRNAs were detected and identified through the utilization of find_circ1[5] and CIRI2[6]. The initial normalization of the raw counts was accomplished through the utilization of the TPM Normalized expression level = (readCount*1,000,000) / libsize (libsize is the sum of circRNA readcount). For differential expression analysis between two conditions or groups, the DESeq R package (1.24.0) was utilized. The GOseq R package was utilized to conduct Gene Ontology (GO) enrichment analysis on host genes associated with differentially expressed circRNAs.

Tandem Mass Tags (TMT) -based phosphoproteome sequencing

Cells or tissues were subjected to lysis in a buffer solution that contained inhibitors for protease and phosphatase. Concentration of protein was determined through the BCA assay (ThermoFisher Scientific, USA). Proteins were reduced, alkylated, and digested with trypsin. The enrichment of phosphopeptides was achieved through a synergistic application of strong

cation exchange chromatography and titanium dioxide chromatography. Phosphopeptides were desalted using C18 StageTips. The TMT reagents were utilized to label desalted phosphopeptides in accordance with the instructions provided by the manufacturer. Labeled peptides were amalgamated and separated through high-pH reverse-phase chromatography. Fractions were concatenated into 6 samples for LC-MS/MS analysis. The process of peptide separation was conducted through reverse-phase chromatography, employing an EASY-nLC 1200 system (ThermoFisher Scientific, USA). Peptides were analyzed on a Q Exactive HF-X mass spectrometer (ThermoFisher Scientific, USA). Data was searched against a protein database using a search engine such as MaxQuant. Phosphorylation sites were quantified using the TMT reporter ion intensities. Phosphorylation sites were filtered based on localization probability and localization score. Phosphorylation sites were quantified and compared between samples using statistical methods such as t-tests or ANOVA. Pathway analysis was performed to identify enriched signaling pathways and biological processes. The above experimental section was completed by Hangzhou Cosmos Wisdom.

1. Yan Y, Tao H, He J, Huang SY. The HDOCK server for integrated protein-protein docking. *Nature protocols.* 2020;15(5):1829-1852.
2. Zhang Y, Wang J, Xiao Y. 3dRNA: 3D Structure Prediction from Linear to Circular RNAs. *Journal of molecular biology.* 2022;434(11):167452.
3. Jumper J, Evans R, Pritzel A, et al. Highly accurate protein structure prediction with AlphaFold. *Nature.* 2021;596(7873):583-589.
4. Yan Y, Zhang D, Zhou P, Li B, Huang SY. HDOCK: a web server for protein-protein

and protein-DNA/RNA docking based on a hybrid strategy. *Nucleic acids research.* 2017;45(W1):W365-w373.

5. Memczak S, Jens M, Elefsinioti A, et al. Circular RNAs are a large class of animal RNAs with regulatory potency. *Nature.* 2013;495(7441):333-338.

6. Gao Y, Zhang J, Zhao F. Circular RNA identification based on multiple seed matching. *Briefings in bioinformatics.* 2018;19(5):803-810.

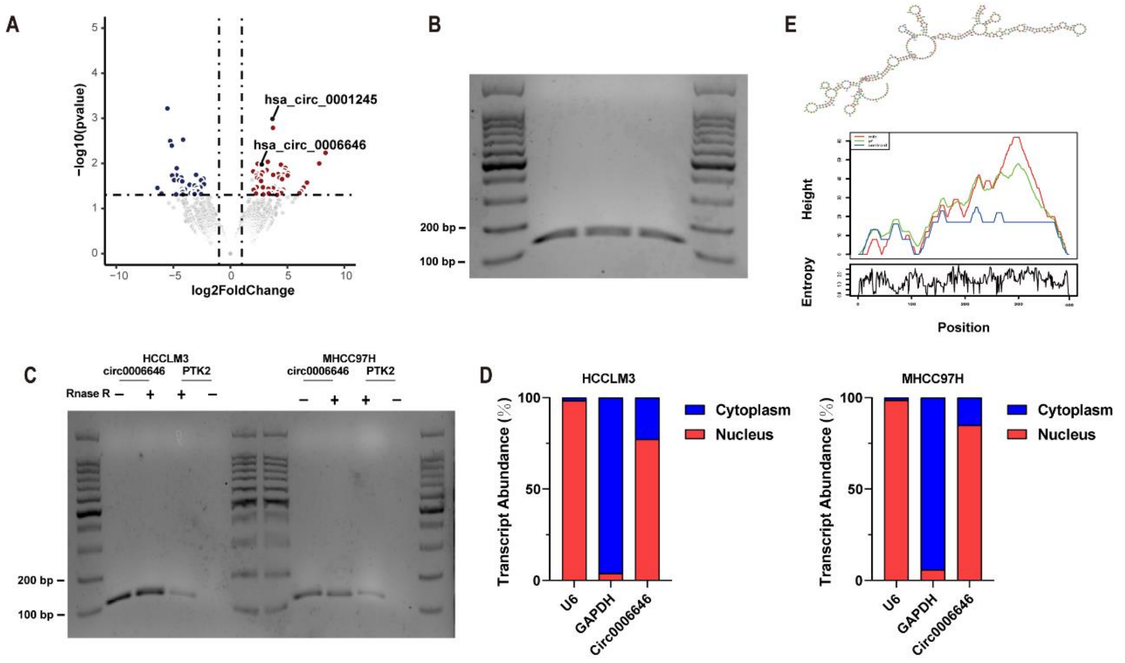

Supplementary figure 1 Circ0006646 was more stable than its parent gene PTK2

(A) volcano plot showed the up-regulated and down-regulated circRNAs. (B) Agarose gel electrophoresis was performed to show the products of PCR using divergent primer. (C) Total RNA digested with and without RNase R was amplified with primers of circ0006646 and PTK2, respectively, followed by agarose gel electrophoresis. (D) Subcellular distribution of circ0006646 was determined by qPCR with nuclear or cytoplasmic fractionation. GAPDH and U6 were chosen for cytoplasmic and nuclear internal references, respectively. (E) RNAfold predicted the secondary structure of circ0006646 with minimum free energy.

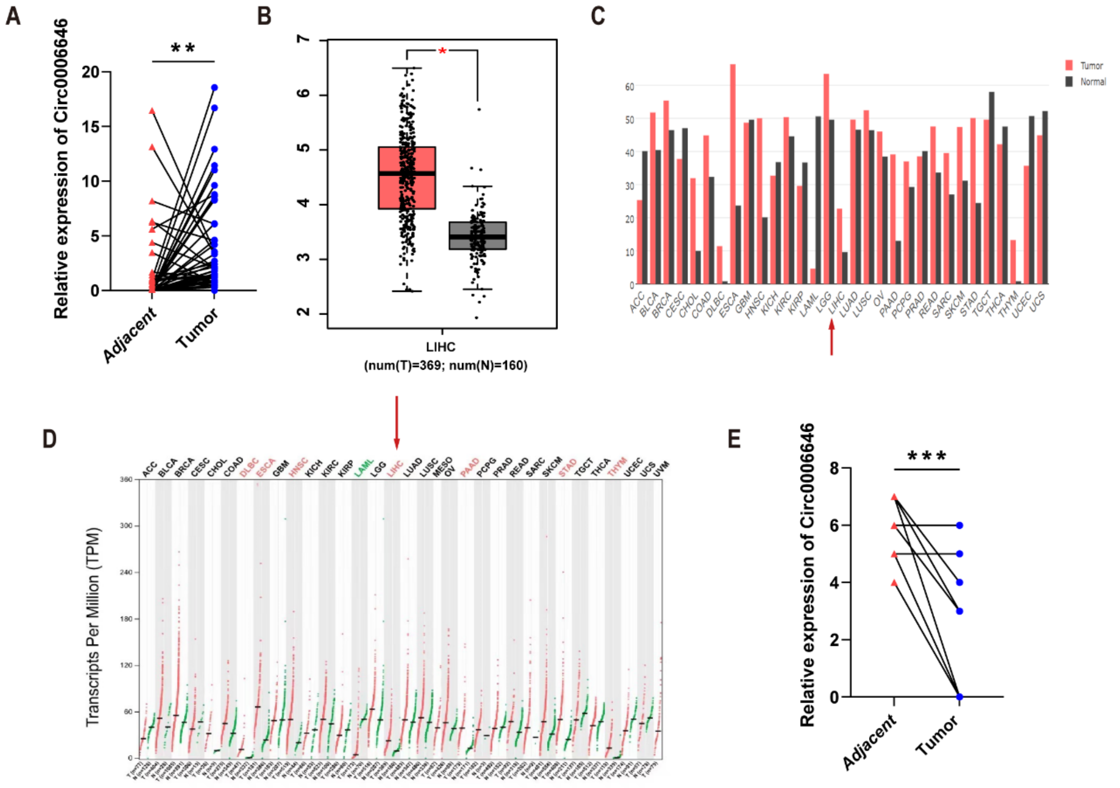

Supplementary figure 2 PTK2 was upregulated in HCC, but did not affect prognosis

(A) The expression level of circ0006646 in paired tumor and adjacent tissues was detected by qRT-PCR (HCC patients from cohort 1, n = 45). (B-D) Gene Expression Profiling Interactive Analysis (GEPIA) database (http://gepia.cancer-pku.cn/) was used to analyze the expression level of PTK2. (E) ISH scores of tumor and adjacent tissues were shown (HCC patients from cohort 1, n = 10). **p < 0.01, ***p < 0.001.

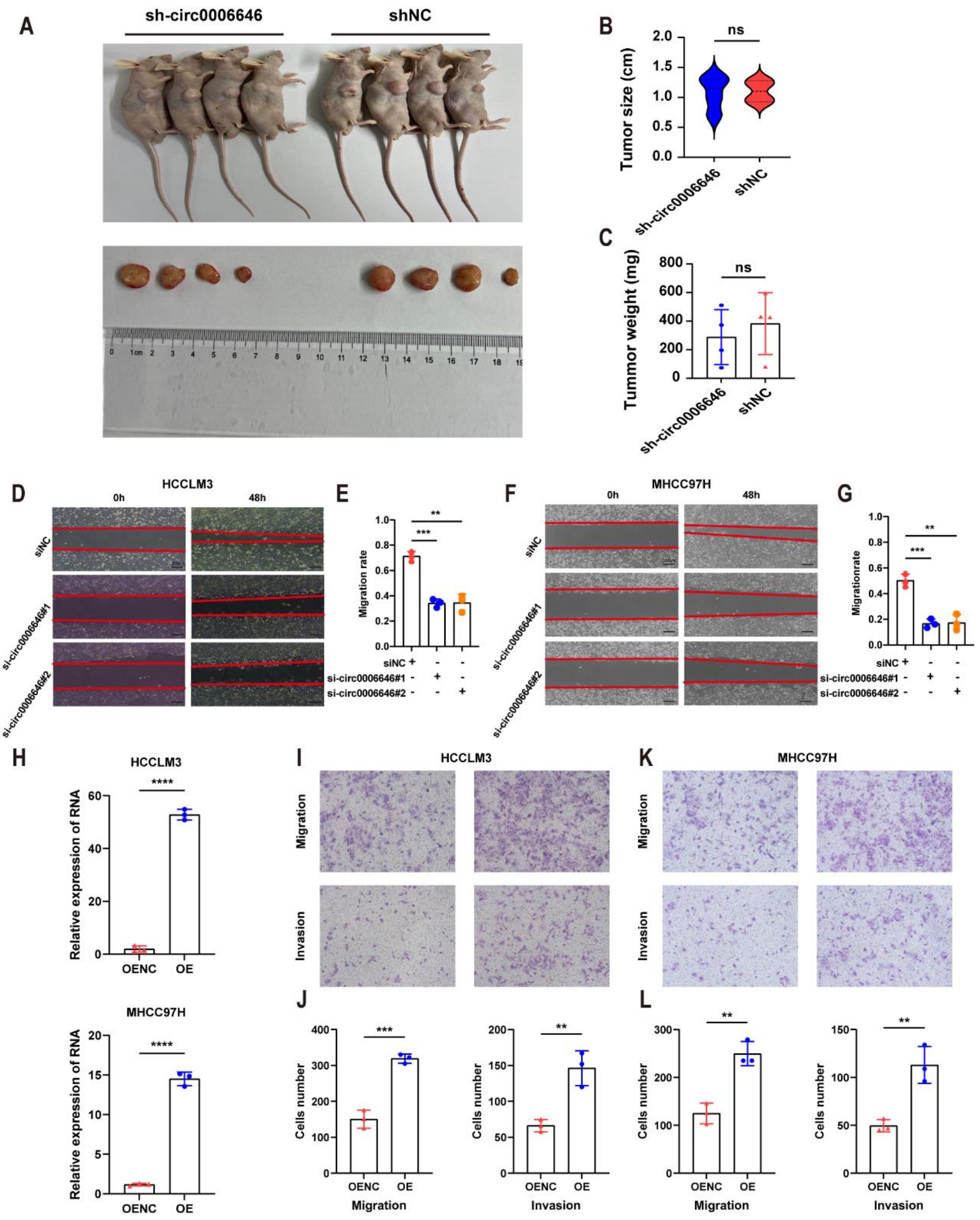

Supplementary figure 3 Circ0006646 inhibited migration but had no effect on the proliferation of HCC cells.

(A-C) HCCLM3$^{circ0006646\text{-}low}$ or HCCLM3$^{circ0006646\text{-}high}$ were implanted subcutaneously into mice. After 4 weeks, the growth of tumors in vivo was observed (A). Subcutaneous tumors of mice were obtained and tumor size (B) and number (C) were analyzed, n = 4 mice/group. (D-G) After cells were transfected with siNC and si-circ0006646, the migration ability of HCCLM3

(D-E) and MHCC97H (F-G) were evaluated using wound-healing assay. Scale bars, 50μm. (H) After cells were transfected with OE-NC and OE-circ0006646, the expression level of circ0006646 and PTK2 in HCCLM3 and MHCC97H were detected by qRT-PCR. (I-L) After cells were transfected with OE-NC and OE-circ0006646, transwell assay was used to evaluate the migration and invasion ability of HCCLM3 (I-J) and MHCC97H (K-L). $^{**}p < 0.01$, $^{***}p < 0.001$.

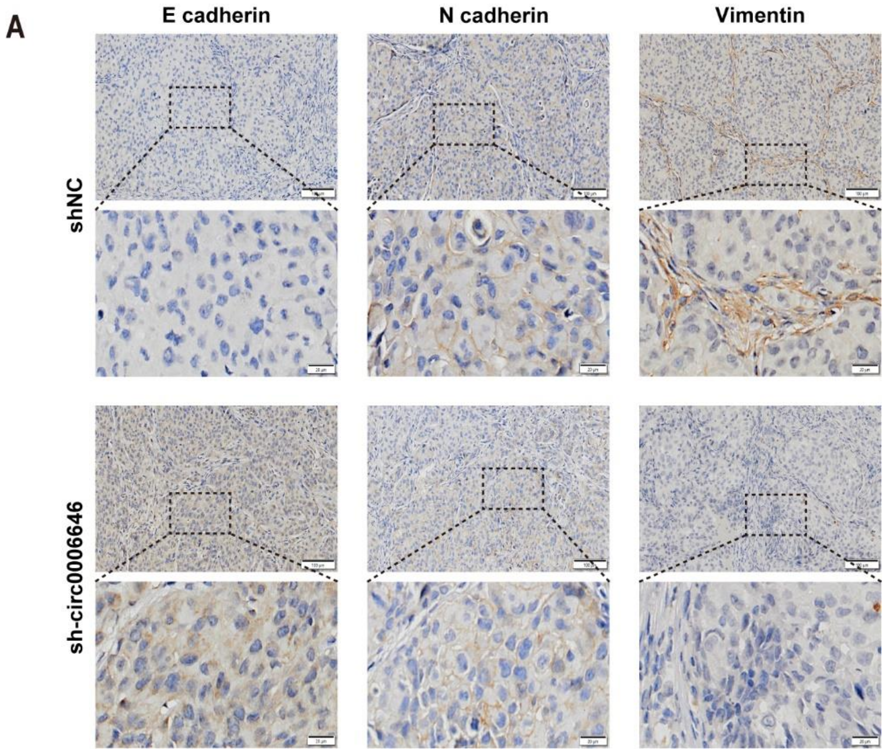

Supplementary figure 4 Circ0006646 silencing inhibited EMT activation

(A) Representative ISH staining of E cadherin, N cadherin and vimentin of subcutaneous tumors in HCCLM3$^{circ0006646\text{-}low}$ group and HCCLM3$^{circ0006646\text{-}high}$ group. Scale bars, 100μm and 20μm, respectively.

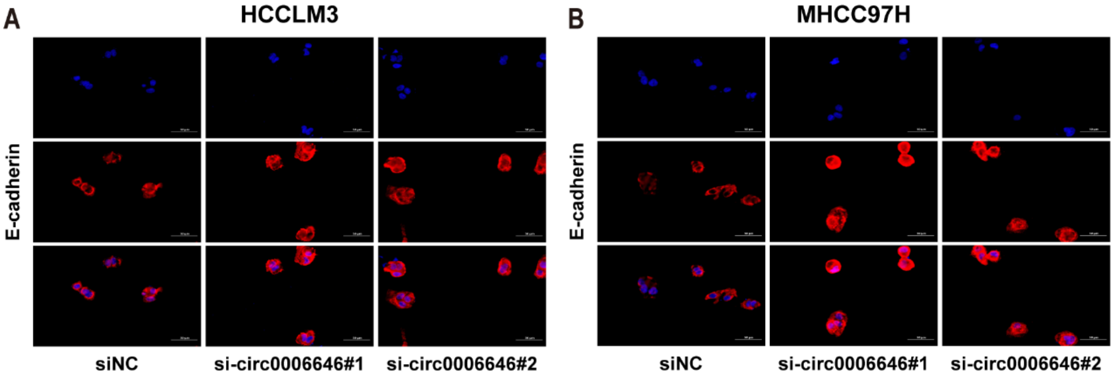

Supplementary figure 5 Circ0006646 silencing inhibited EMT activation

(A-B) After cells were transfected with siNC and si-circ0006646, representative IF images of E cadherin HCCLM3 (A) and MHCC97H (B) were shown. DAPI staining represented the location of the nucleus. Scale bars, 50μm

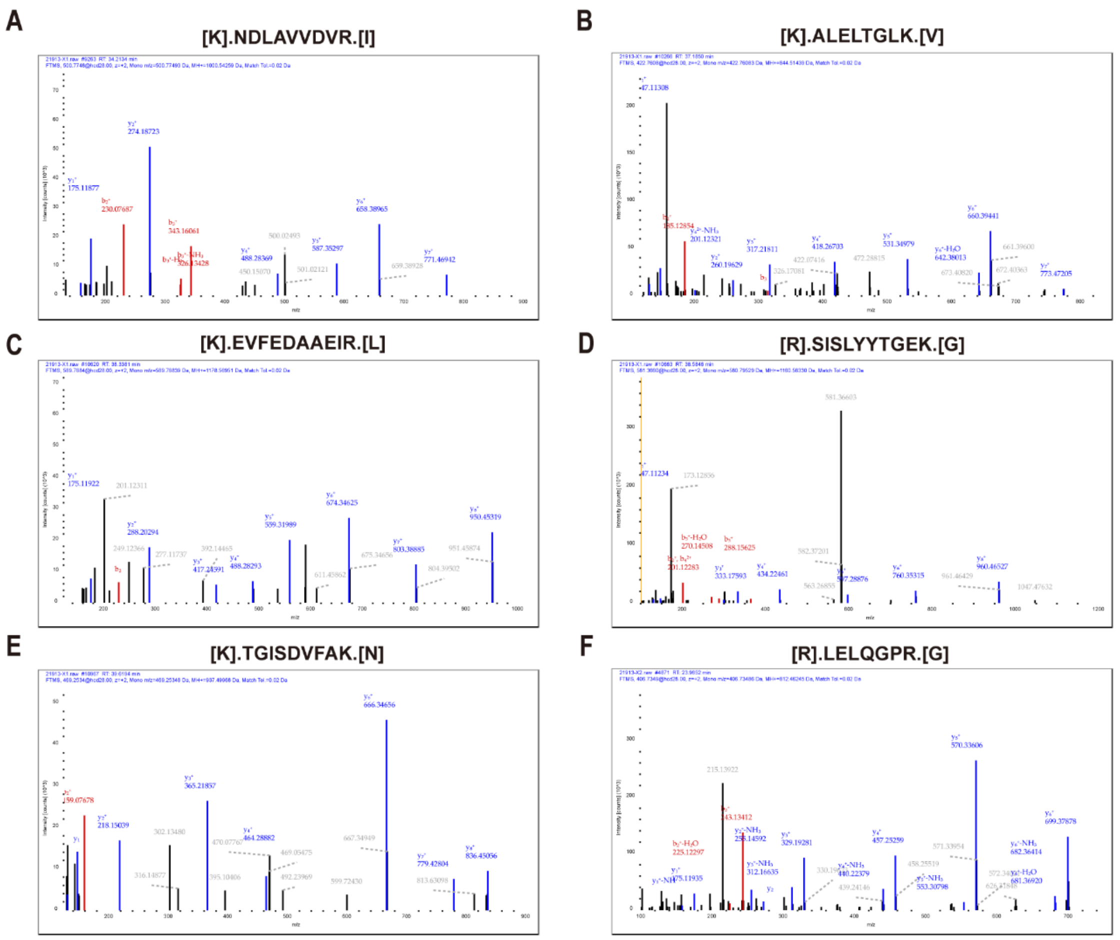

Supplementary figure 6 NCL peptides identified by mass spectrometry

(A-F) NCL peptides enriched by 2 circ0006646 specific probes in mass spectrometry were shown.

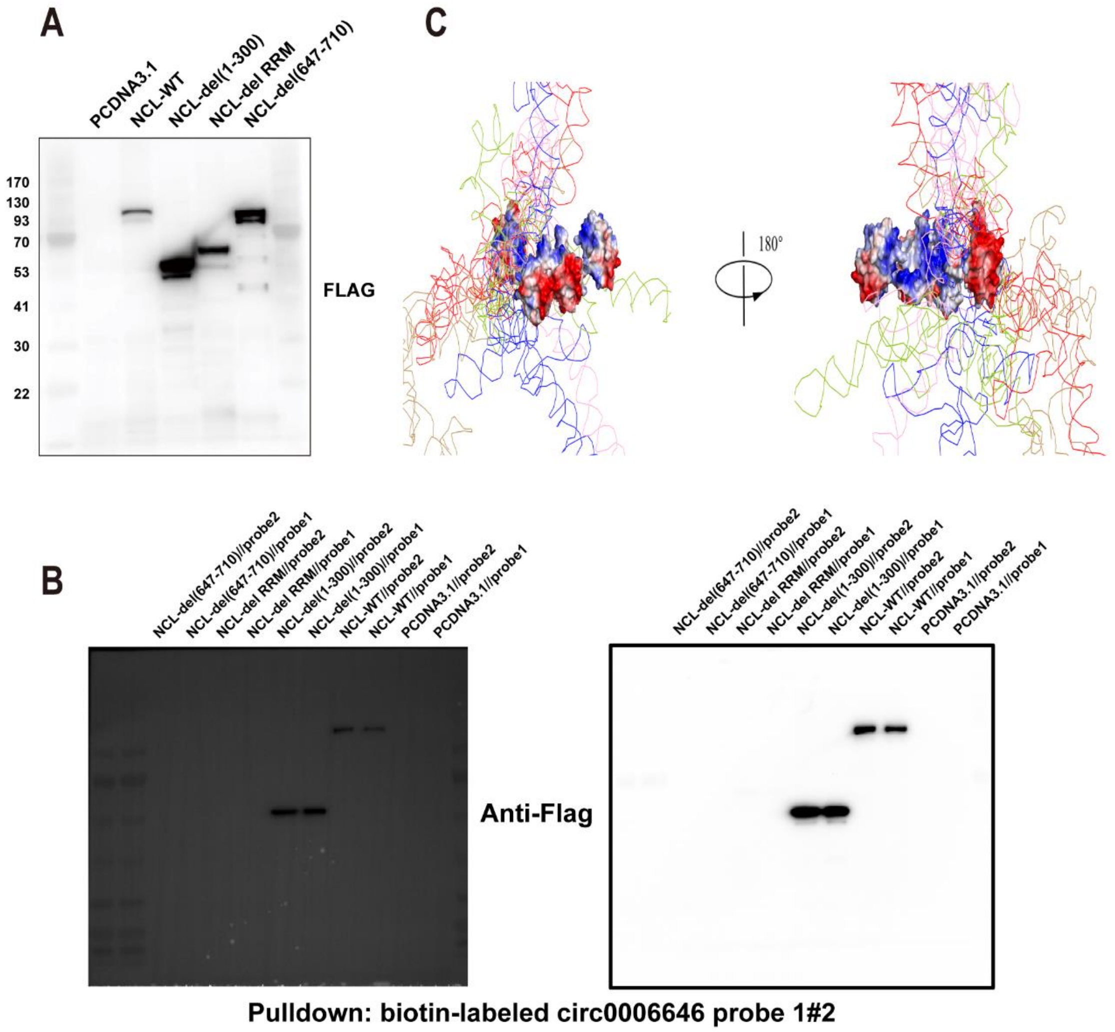

Supplementary figure 7 Structural prediction of the interaction of circ0006646 and NCL

(A) The expression of proteins after transfection with NCL wild type and truncated mutant plasmid were verified. Blots with antibodies recognizing the FLAG was show. (B) 293T cells were transfected with NCL-FlAG, NCL del (1-300) FlAG, NCL del (RRM) FlAG, NCL del (647-710) Flag plasmid. After cell lysis using RIPA, circ0006646 specific targeting probe were used for pulldown assay. Blots with antibodies recognizing the FLAG were shown. (C) The top five docking poses of the circRNAs onto NCL protein were shown. The protein was represented by an electrostatic potential surface (blue: positively charged, red: negatively charged). The RNA backbones were shown in line representation with one color indicating a particular pose.

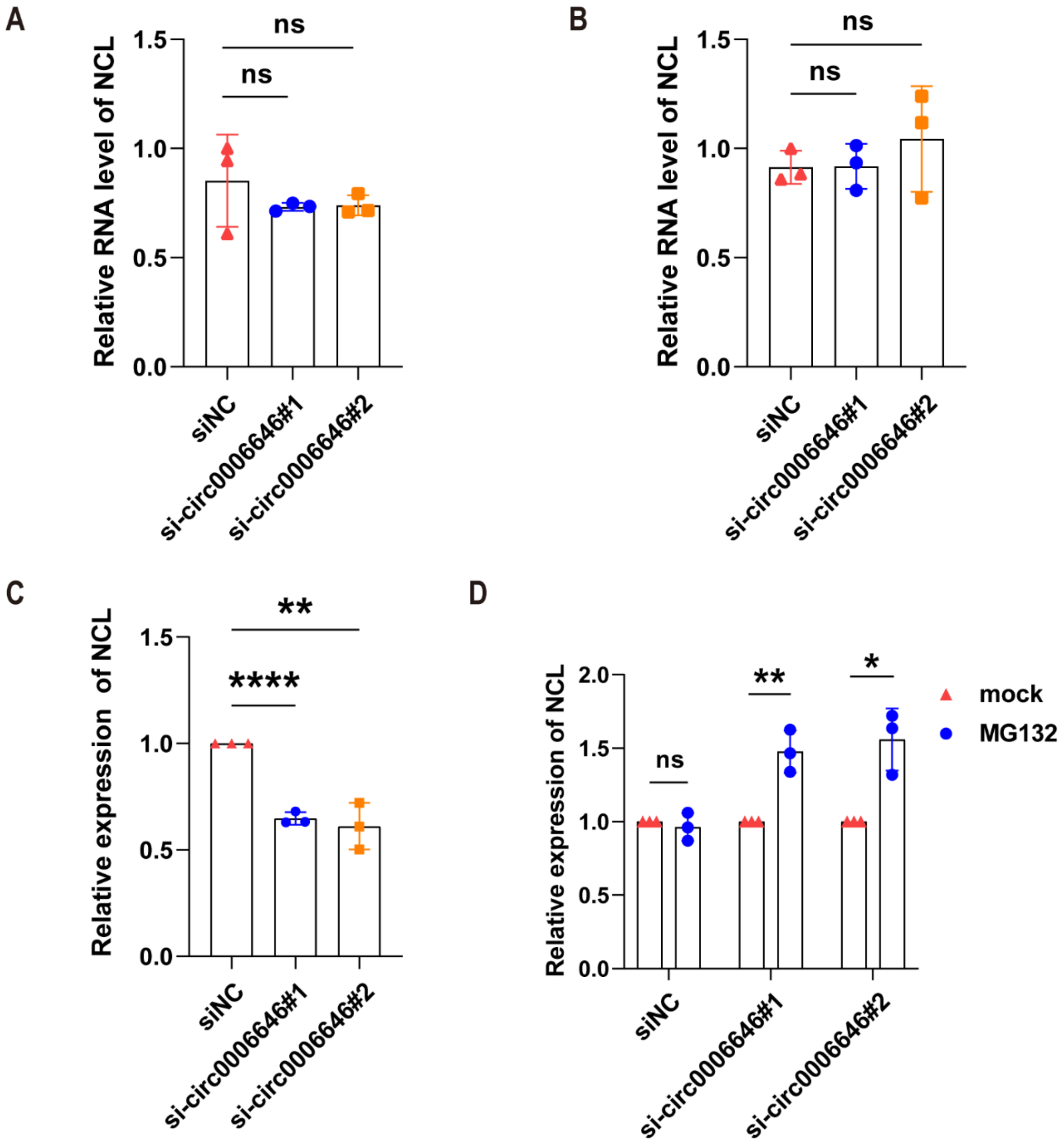

Supplementary figure 8 Circ0006646 stabilizes NCL expression at the protein translation level

(A-B) After cells were transfected with siNC and si-circ0006646, the mRNA content of NCL in HCCLM3 (A) and MHCC97H (B) were evaluated by qRT-PCR. (C) After HCCLM3 was transfected with siNC and si-circ0006646, the expression change of NCL was statistically quantified. (D) HCCLM3 was transfected with siNC and si-circ0006646, and later treated with 10 μM MG132 for 6h. The expression change of NCL was statistically quantified. *$p < 0.05$, **$p < 0.01$, ***$p < 0.001$, ****$p < 0.0001$.

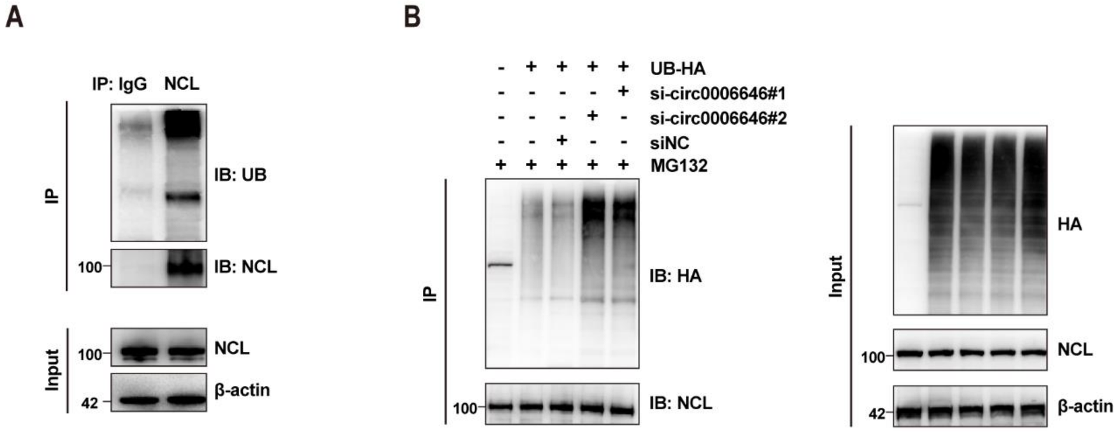

Supplementary figure 9 Circ0006646 inhibited the ubiquitination of NCL

(A) Co-IP assay was performed. The HCCLM3 lysate was incubated with anti-NCL and anti-IgG, and later incubated with protein A/G magnetic beads. Blots with antibodies recognizing the UB and NCL were shown. 5% lysate was used as the input control. Blots with antibodies recognizing the NCL and β-Actin were shown. (B) Co-IP assay was performed. 293T cells transfected with HA-tagged UB, siNC and si-circ0006646 were treated with 10 μM MG132 for 6h. The 293T lysate was incubated with anti-NCL, and later incubated with protein A/G magnetic beads. Blots with antibodies recognizing the HA and NCL were shown. 5% lysate was used as the input control. Blots with antibodies recognizing the HA, NCL and β-Actin were shown.

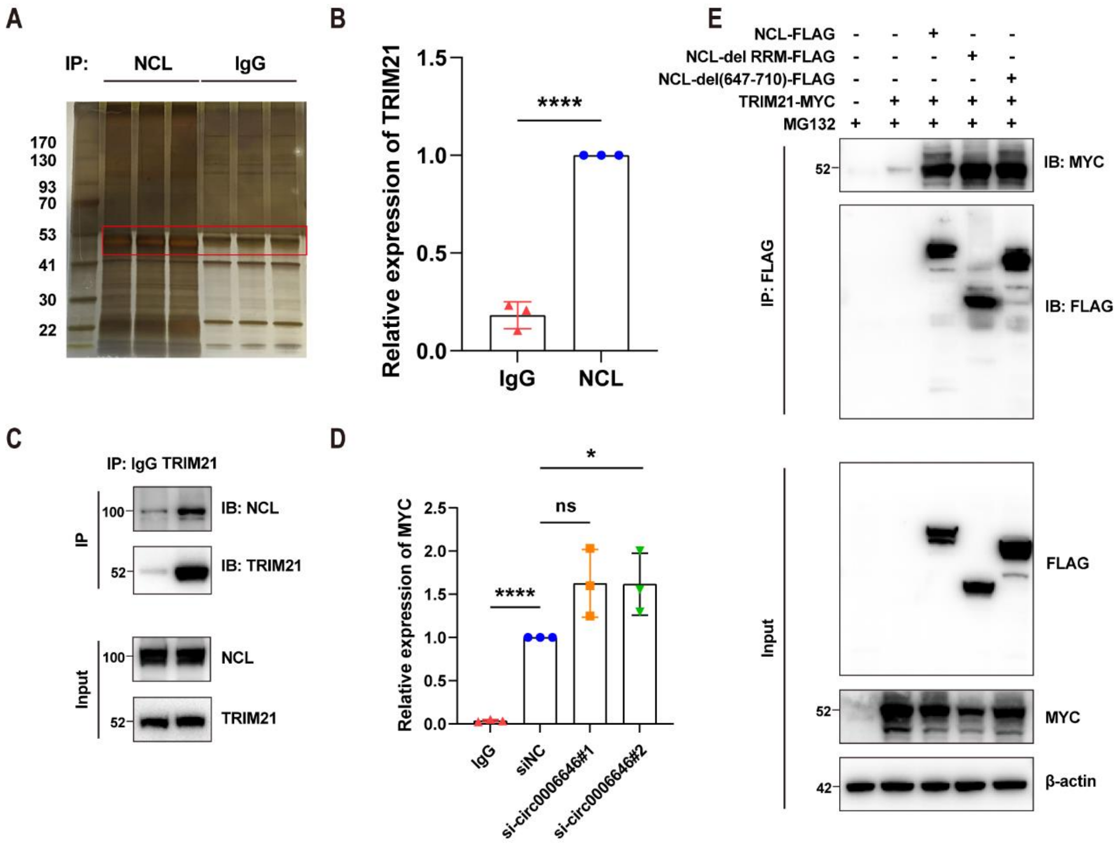

Supplementary figure 10 Circ0006646 inhibited the binding of NCL to TRIM21

(A) Co-IP experiments were performed with anti-NCL and anti-IgG, and the obtained protein samples were then silver stained. (B) The relative expression of TRIM21 was statistically quantified. (C) The HCCLM3 lysate was incubated with anti-TRIM21 and anti-IgG, and later incubated with protein A/G magnetic beads. Blots with antibodies recognizing the TRIM21 and NCL were shown. (D) The relative expression of MYC was statistically quantified. *$p < 0.05$, ****$p < 0.0001$. (E) 293T cells transfected with TRIM21-MYC, NCL-FlAG, NCL del (RRM) FlAG, NCL del (647-710) FlAG plasmid. The 293T lysate was incubated with anti-NCL, and later incubated with protein A/G magnetic beads. Blots with antibodies recognizing the MYC and FLAG were shown. *$p < 0.05$, ****$p < 0.0001$.

A

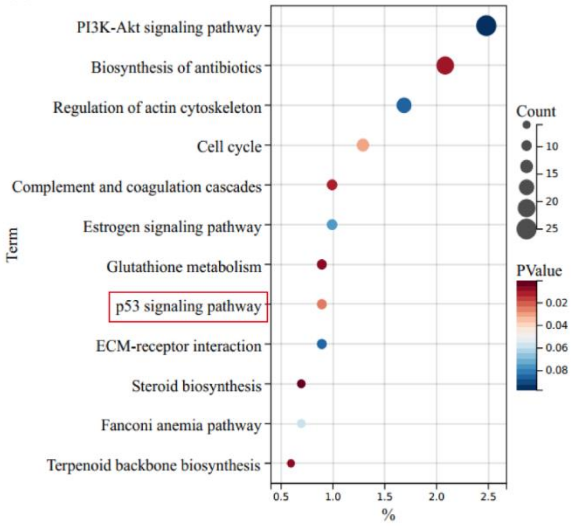

Supplementary figure 11 The p53 pathway was the downstream regulatory mechanism of NCL

(A) Enrichment pathways of transcriptome sequencing in HCCLM3$^{circ0006646\text{-}high}$ and HCCLM3$^{circ0006646\text{-}low}$.

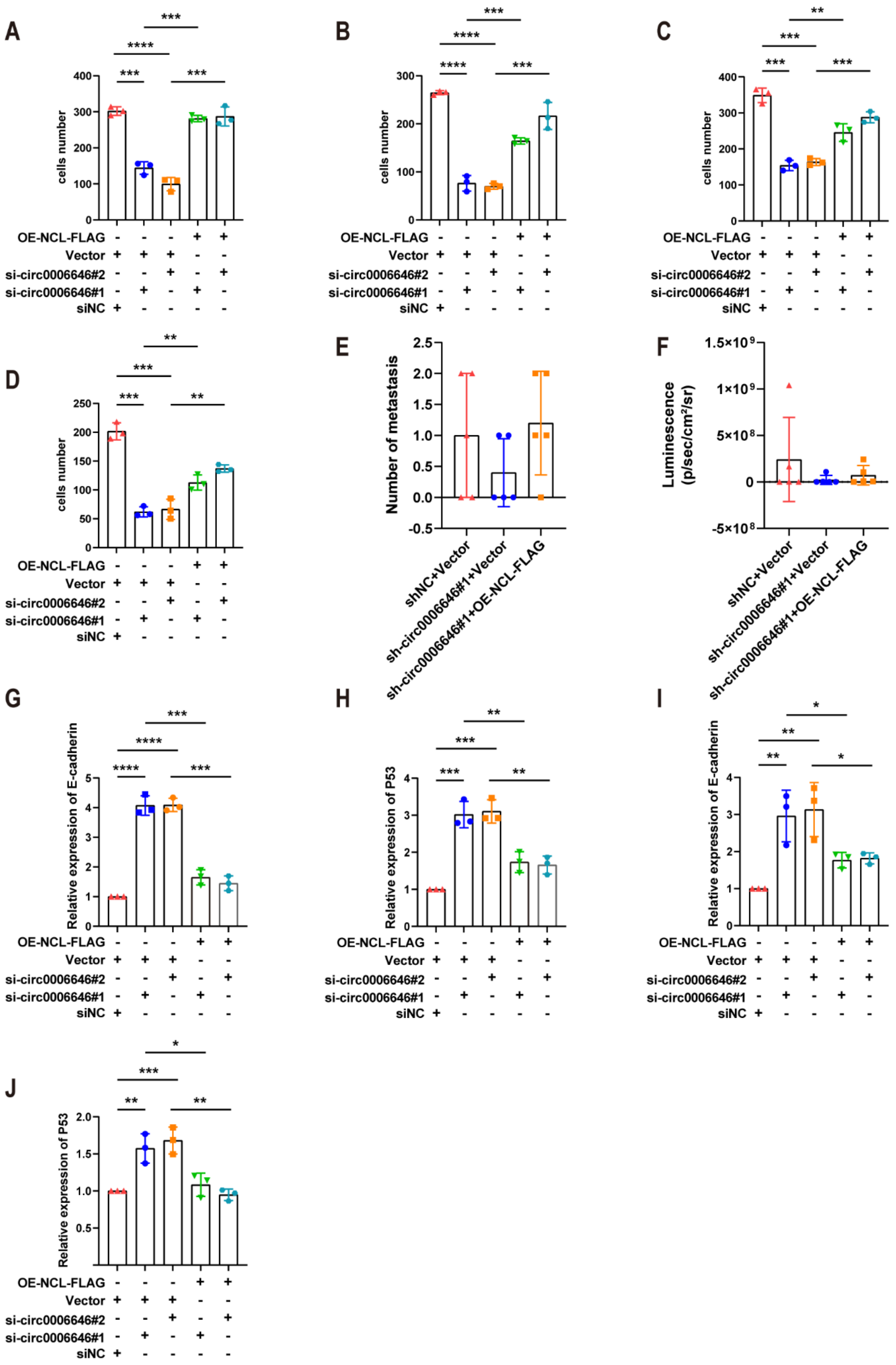

Supplementary figure 12 NCL overexpression rescued the impaired HCC metastasis capacity caused by circ0006646 depletion

(A-D) The numbers of HCCLM3 (A-B) and MHCC97H (C-D) passing through the transwell membrane were statistically quantified. (E-F) The number of liver metastases (E) and the fluorescence intensity in liver metastasis model (F) were compared, n = 5 mice/group. (G-J) Cells were transfected with siNC and si-circ0006646, and later transfected with FLAG tagged OE-NCL plasmid or vector. Relative expression of E cadherin and p53 in HCCLM3 (G-H) and MHCC97H (I-J) were statistically quantified. *$p < 0.05$, **$p < 0.01$, ***$p < 0.001$, ****$p < 0.0001$.

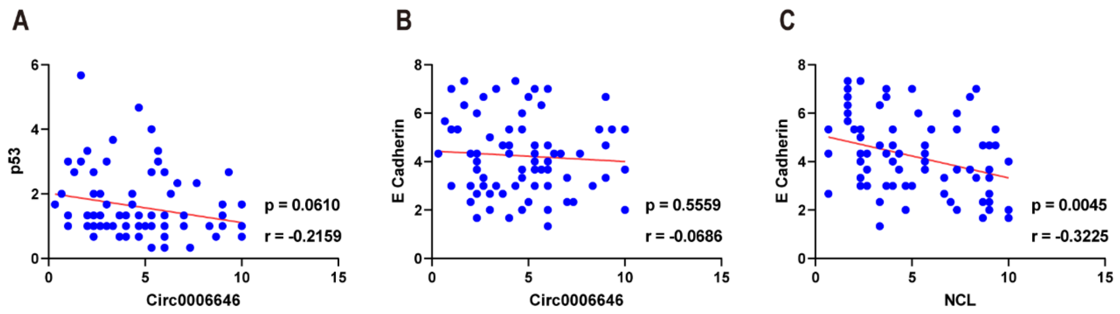

Supplementary figure 13 Expression correlation of circ0006646, NCL, p53 and E cadherin in HCC patients

(A-C) The expression correlations between circ0006646 and p53 (A), circ0006646 and E cadherin (B) and NCL and E cadherin (C) were evaluated (cohort 1, n = 76).

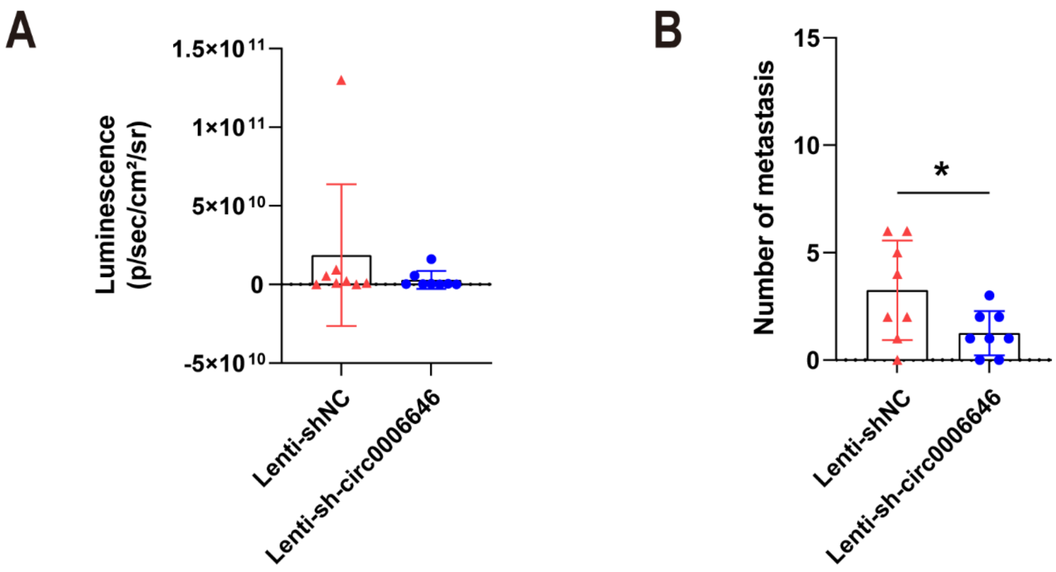

Supplementary figure 14 Systemic therapy targeting circ0006646 inhibited HCC metastasis

(A-B) Fluorescence intensity (A) and number of metastases (B) of mice injected with shNC or sh-circ0006646 lentivirus were analyzed statistically, n = 8 mice/group. $^*p < 0.05$.

Supplementary table 1. Primer sets used for qRT-PCR.

Gene names		Primer sequence (5' to 3')
hsa_circ_0006646	Forward	CCACCAGAGGAGTGGAAAAT
	Reverse	ACTCGAATTTGGTGTGTGATTC
PTK2	Forward	CGAGAGATTGAGATGGCACA
	Reverse	TACTCTTGCTGGAGGCTGGT
hsa_circ_0001245	Forward	AAACAGCAAAGCCCAGGTC
	Reverse	CCGTGTAGAGGCCGTGAAT
TP53-5'UTR	Forward	AAGTCTAGAGCCACCGTCCA
	Reverse	GTGTCACCGTCGTGGAAAG
NCL	Forward	GCACCTGGAAAACGAAAGAAGG
	Reverse	GAAAGCCGTAGTCGGTTCTGT
TP53	Forward	GAGGTTGGCTCTGACTGTACC
	Reverse	TCCGTCCCAGTAGATTACCAC
β-Actin	Forward	TTTGAATGATGAGCCTTCGTGCCC
	Reverse	GGTCTCAAGTCAGTGTACAGGTAAGC
U6	Forward	GCTTCGGCAGCACATATACTAAAAT
	Reverse	CGCTTCACGAATTTGCGTGTCAT
GAPDH	Forward	GGAGCGAGATCCCTCCAAAAT
	Reverse	GGCTGTTGTCATACTTCTCATGG
hsa_circ_0006646-OE	Forward	CCCACCAGAGGAATGGAAGT
	Reverse	CGAATTTGGTGTGTGATTCAA

qRT-PCR, quantitative reverse transcription polymerase chain reaction; UTR, untranslated region; NCL, nucleolin.

Supplementary table 2. Antibodies used in the study.

Name	Citation (PMID)	Supplier	Cat no.
E cadherin	**36828547**	Cell Signaling Technology	#3195
N cadherin	**36582605**	Cell Signaling Technology	#13116
vimentin	**34012073**	Proteintech	10366-1-AP
Nucleolin	**34078433**	Abcam	ab129200
Nucleolin	**24598541**	Proteintech	10556-1-AP
β-Actin	**32581380**	Proteintech	66009-1-Ig
p53	**35210429**	Abcam	ab32389
Ubiquitin	**36632468**	Cell Signaling Technology	#3936
Ubiquitin (linkage-specific K48)	**33393215**	Abcam	ab140601
Anti-Ubiquitin (linkage-specific K63)	**33393215**	Abcam	ab179434
HA-tag	**33531476**	Abcam	ab1424
Flag-tag	**36604149**	Cell Signaling Technology	#14793
Myc tag	**33053435**	Abcam	ab9106
TRIM21	**36692217**	Proteintech	67136-1-Ig
IgG	**35585044**	Abcam	ab172730
Twist2	**36111260**	Affinity	AF4112

Supplementary table 3. Sequences for the nucleic acids.

Target	Targeted Sequence (5'-3')
circ0006646 FISH probe	GGTATCTGTCATATTTTCCACTCCTCTGGT
circ0006646 pulldown probe1	GTATCTGTCATATTTTCCACTCCTCTGG
circ0006646 pulldown probe2	GGTATCTGTCATATTTTCCACTCCTCTGGT
circ0006646 ISH probe	GGTATCTGTCATATTTTCCACTCCTCTGGT
circ0006646 shRNA	CCAGAGGAGTGGAAAATATGACAGA
circ0006646 siRNA 1	CCAGAGGAGTGGAAAATATGACAGA
circ0006646 siRNA 2	AGAGGAGUGGAAAAUAUGACAGAUA

FISH, fluorescence in situ hybridization; ISH, in situ hybridization.

Supplementary table 4. The differential expression circRNAs.

CircRNA_ ID	mvi_no1.re adcount	mvi_no2.re adcount	mvi_no3.re adcount	mvi_no4.re adcount	mvi_no5.re adcount	mvi_yes1.re adcount	mvi_yes2.re adcount	mvi_yes3.re adcount	mvi_yes4.re adcount	mvi_yes5.re adcount
hsa_circ_0 001259	0	0	0	0	0	17	12	3	0	0
hsa_circ_0 000339	0	0	0	0	0	367	0	0	0	20
hsa_circ_0 008030	0	9	3	0	0	22	16	3	4	9
hsa_circ_0 025544	0	0	0	0	0	17	11	0	0	3
hsa_circ_0 007731	0	0	0	0	0	8	12	8	0	0
hsa_circ_0 001245	4	7	2	0	0	91	19	4	5	12
hsa_circ_0 039351	0	9	3	0	0	38	4	4	4	7
hsa_circ_0 075829	0	0	0	0	0	30	5	0	0	3
hsa_circ_0 002141	38	31	55	0	0	767	488	164	0	93
hsa_circ_0 080301	0	0	0	0	0	3	5	8	0	2
hsa_circ_0 061776	0	0	0	0	0	39	5	0	0	0
hsa_circ_0	0	0	0	0	0	3	0	0	0	7

066187										
hsa_circ_0002778	0	0	0	0	0	11	17	10	0	0
hsa_circ_0005366	0	4	0	0	0	24	4	0	0	16
hsa_circ_0008942	0	0	0	0	0	9	13	0	0	5
hsa_circ_0047698	0	0	0	0	0	3	5	6	3	0
hsa_circ_0047690	0	0	0	0	0	6	0	0	5	0
hsa_circ_0001180	0	0	0	0	0	8	0	0	7	0
hsa_circ_0032203	0	0	0	0	0	11	0	21	0	0
hsa_circ_0003130	0	0	0	0	0	10	11	10	0	0
hsa_circ_0084668	0	0	2	0	0	17	9	0	6	0
hsa_circ_0078735	0	0	0	0	0	26	0	0	0	12
hsa_circ_0000885	0	0	0	0	0	20	0	0	0	9
hsa_circ_0066568	2	17	9	0	0	177	16	0	5	5
hsa_circ_0	0	0	0	0	0	11	9	0	2	0

060461										
hsa_circ_0004603	0	0	0	0	0	14	7	0	0	6
hsa_circ_0016336	0	0	0	0	0	3	0	0	0	7
hsa_circ_0006646	8	48	27	0	0	296	60	6	7	24
hsa_circ_0006126	0	0	0	0	0	3	0	0	0	9
hsa_circ_0010203	0	0	0	0	0	22	19	0	0	2
hsa_circ_0001356	2	0	0	0	0	5	0	0	15	8
hsa_circ_0004445	0	0	0	0	0	14	6	9	0	0

Inclusion criteria: 1) log2-fold change >1.5 or < -1.5, 2) upregulated in MVI-present tissues, 3) p value < 0.02.

Supplementary table 5. Univariate and Multivariate analyses.

	Univariate analysis		Multivariate analysis	
Variables	HR (95%CI)	*p* value	HR (95%CI)	*p* value
Circ0006646 expression	9.020 (1.191-68.331)	0.033	8.902(1.149-68.986)	0.036
Gender	0.038 (0.000-14.053)	0.279		
Age	1.498 (0.558-4.025)	0.423		
Grade stage	3.726 (1.383-10.036)	0.009	3.274 (1.202-8.919)	0.020
Tumor size	2.987 (1.111-8.032)	0.030	3.253 (1.151-9.193)	0.019
T stage	0.988 (0.343-2.844)	0.982		
TNM stage	1.210 (0.439-3.330)	0.712	0.965 (0.330-2.825)	0.948

HR, Hazard Ratio; CI, Confidence Interval

Supplementary table 6. Top 10 terms in GO Enrichment Analysis of TMT-based phosphoproteomics sequencing.

No.	Term	Count	%	PValue	List Total	Pop Hits	Pop Total	Fold Enrichment	Bonferroni	Benjamini	FDR
1	GO:0016477~cell migration	23	5.651106	6.07E-08	395	278	19453	4.074483	1.51E-04	1.52E-04	1.50E-04
2	GO:0006364~rRNA processing	14	3.439803	4.38E-06	395	137	19453	5.032653	0.010873	0.005466	0.005425
3	GO:0051301~cell division	23	5.651106	1.26E-05	395	383	19453	2.957458	0.03104	0.010511	0.010431
4	GO:0042274~ribosomal small subunit biogenesis	10	2.457002	1.81E-05	395	74	19453	6.655149	0.044142	0.011286	0.0112
5	GO:0008380~RNA splicing	16	3.931204	2.79E-05	395	211	19453	3.734453	0.067151	0.013902	0.013796
6	GO:0098609~cell-cell adhesion	15	3.685504	4.61E-05	395	195	19453	3.788315	0.108598	0.017409	0.017277
7	GO:0098911~regulation of ventricular cardiac muscle cell action potential	5	1.228501	4.89E-05	395	11	19453	22.3855	0.114735	0.017409	0.017277
8	GO:0045445~myoblast differentiation	6	1.474201	6.59E-05	395	22	19453	13.4313	0.151463	0.02053	0.020373
9	GO:0030036~actin cytoskeleton organization	16	3.931204	8.26E-05	395	232	19453	3.396421	0.186234	0.022897	0.022723
10	GO:0006887~exocytosis	11	2.702703	1.17E-04	395	115	19453	4.710688	0.252773	0.029137	0.028915
